# Multigene manipulation of photosynthetic carbon assimilation increases CO_2_ fixation and biomass yield in tobacco

**DOI:** 10.1093/jxb/erv204

**Published:** 2015-05-08

**Authors:** Andrew J. Simkin, Lorna McAusland, Lauren R. Headland, Tracy Lawson, Christine A. Raines

**Affiliations:** School of Biological Sciences, Wivenhoe Park, University of Essex, Colchester CO4 3SQ, UK

**Keywords:** Biomass, Calvin–Benson cycle, chlorophyll fluorescence imaging, gas exchange, gene stacking.

## Abstract

Over the next 40 years it has been estimated that a 50% increase in the yield of grain crops such as wheat and rice will be required to meet the food and fuel demands of the increasing world population. Transgenic tobacco plants have been generated with altered combinations of sedoheptulose-1,7-bisphosphatase, fructose-1,6-bisphosphate aldolase, and the cyanobacterial putative-inorganic carbon transporter B, ictB, of which have all been identified as targets to improve photosynthesis based on empirical studies. It is shown here that increasing the levels of the three proteins individually significantly increases the rate of photosynthetic carbon assimilation, leaf area, and biomass yield. Furthermore, the daily integrated measurements of photosynthesis showed that mature plants fixed between 12–19% more CO_2_ than the equivalent wild-type plants. Further enhancement of photosynthesis and yield was observed when sedoheptulose-1,7-bisphosphatase, fructose-1,6-bisphosphate aldolase, and ictB were over-expressed together in the same plant. These results demonstrate the potential for the manipulation of photosynthesis, using multigene-stacking approaches, to increase crop yields.

## Highlight

Multigene manipulation of levels of Calvin cycle enzymes, together with the introduction of a putative cyanobacterial inorganic carbon transporter, results in substantial improvements in biomass yield. This study demonstrates that this approach has the potential to produce crop plants to meet the food requirements of a growing population.

## Introduction

Increasing demands of the growing world population for food and fuel are putting ever greater pressure on the need to develop higher-yielding crop varieties. It has been estimated that increases of 50% will be required in the yield of grain crops such as wheat and rice if food supply is to meet the demands of the increasing world population (Fischer and Edmeades, 2010). The maximum yield of a crop is determined by the yield potential, which is the biomass produced per unit area of land over the growing season under optimal conditions and is influenced by genetic factors and agronomic practice. The primary determinant of crop yield is the cumulative rate of photosynthesis over the growing season which is the result of the crop’s ability to capture light, the efficiency by which this light is converted to biomass, and how much biomass is converted into the usable product, for example, grain in the case of wheat and rice. Traditional breeding and agronomic approaches have maximized light capture and the conversion of biomass to end-products and, therefore, in order to increase yield, the efficiency of energy conversion will have to be improved (Zhu *et al.*, 2010). In plants that fix atmospheric CO_2_ using the Calvin–Benson (C3) cycle enzyme, ribulose-1,5-bisphosphate carboxylase, the theoretical maximum energy conversion efficiency attainable is 4.6%, but, in the field, efficiencies of less than 50% of this are realized. Kinetic models based on ordinary differential equations (ODEs) have been developed to describe the responses of photosynthetic carbon assimilation (Peterson and Ryde-Peterson, 1988; Laisk *et al.*, 1989; Poolman *et al.*, 2000). Further development of these models to include not only the reactions in the Calvin cycle but, importantly, those in the pathways of sucrose and starch biosynthesis and photorespiration has led to the construction of a dynamic model of carbon metabolism (Zhu *et al.*, 2007). The outputs of this modelling work suggested that an increase in the Calvin cycle enzymes sedoheptulose-1,7-bisphosphatase (SBPase: EC.3.1.3.37) and fructose-1,6-bisphosphate aldolase (FBPA: EC 4.1.2.13) and the starch biosynthesis enzyme ADP-glucose pyrophosphorylase (AGPase), together with a decrease in the photorespiratory enzyme glycine decarboxylase (GDC), could increase photosynthetic carbon assimilation.

In addition to these theoretical predictions there is compelling evidence from transgenic studies that manipulation of the C3 cycle will contribute to closing this gap in efficiency and that this could increase yield in the absence of significant stress (Raines, 2006, 2011; Zhu *et al.*, 2010). In the 1990s, analysis of transgenic plants, in which the levels of individual proteins or enzymes were manipulated, changed the view that there was a single limiting step in photosynthetic carbon assimilation (Stitt and Schulze, 1994; Raines, 2003). These studies demonstrated that small reductions in either SBPase or FBPA in the C3 cycle impacted negatively on photosynthesis, indicating that these enzymes had significant control over the rate of carbon assimilation and growth (Haake *et al.*, 1998, 1999; Harrison *et al.*, 1998; Raines *et al.*, 1999; Lawson *et al.*, 2006; Raines and Paul, 2006). These experiments suggested that improvements in photosynthetic carbon fixation may be achieved by increasing the activity of these enzymes individually. Evidence supporting this hypothesis came from transgenic tobacco plants in which the levels of the cyanobacterial SBPase/FBPase (Miyagawa *et al.*, 2001) or the enzyme SBPase (Lefebvre *et al.*, 2005; Rosenthal *et al.*, 2011) were increased. The single manipulation of SBPase resulted in an increase in photosynthesis, leaf area and total biomass was up by as much as 30% in plants grown in high light (Lefebvre *et al.*, 2005). However, growth of SBPase over-expressing plants in greenhouse conditions in the winter, when the day length was shorter and light levels lower, resulted in only minimal increases in growth. Furthermore, growth of these transgenic plants in the field in elevated CO_2_ conditions also resulted in a stimulation of photosynthesis and biomass yield (Rosenthal *et al.*, 2011). It should also be noted that increased levels of SBPase in rice did not improve photosynthesis under non-stress conditions, however, this manipulation helped to maintain photosynthesis when plants were exposed to heat or osmotic stress (Feng *et al.*, 2007*a*, *b*). More recently, the over-expression of FBPA in transgenic tobacco plants resulted in increased photosynthesis and biomass but this was only significant at elevated levels of CO_2_ (Uematsu *et al.*, 2012). In addition to the direct manipulation of the C3 cycle, expression of the putative-inorganic carbon transporter B (*ictB:* YP399376), a gene proposed to be involved in $HCO_{3}^{-}$ accumulation in the cyanobacterium *Synechococcus* sp. PCC 7942 (Bonfil *et al.*, 1998), in *Arabidopsis*, and in tobacco plants resulted in an improvement of photosynthesis and increased biomass when compared with the wild type (Lieman-Hurwitz *et al.*, 2003, 2005). These authors proposed that expression of ictB in plants enhances photosynthesis and growth due to a higher internal CO_2_ concentration around ribulose-1,5-bisphosphate carboxylase/oxygenase (Rubisco) resulting in higher enzyme activity (Lieman-Hurwitz *et al.*, 2003).

The work in this paper aims to test the hypothesis that gene-stacking of components of the C3 cycle with the putative-inorganic carbon transporter B can have a synergistic effect on photosynthesis and yield. To test this, several sets of transgenic tobacco plants co-expressing SBPase and ictB, either alone or in combination, and plants co-expressing SBPase, FBPA, and ictB were generated. It has been shown that the simultaneous manipulation of multiple targets leads to a cumulative impact on photosynthesis and biomass yield which will benefit substantially the biomass requirements of both the biofuel and food industries.

## Materials and methods

### Construct generation

Constructs were generated using Gateway cloning technology and vectors pGWB2 (Nakagawa *et al.*, 2007) and pDESTOE (Booker *et al.*, 2004). Transgenes were under the control of the CaMV 35S and FMV (Richins *et al.*, 1987) constitutive promoters. Construct maps are shown in Supplementary Fig. S1 at *JXB* online. Full details of B2-TB, B2-FB, and FB-TB construct assembly can be seen in the Supplementary Materials and Methods at *JXB* online.

### Generation of transgenic plants

The recombinant plasmids B2-TB, B2-FB, and FB-TB were introduced into wild-type tobacco (*Nicotiana tabacum*) L. cv Samsun or SBPase over-expressing tobacco cv. Samsun (Lefebvre *et al.*, 2005) using *Agrobacterium tumefaciens* AG1 via leaf-disc transformation (Horsch *et al.*, 1985). Shoots were regenerated on MS medium containing kanamycin (100mg l^–1^), hygromycin (300mg l^–1^), and augmentin (500g ml^–1^). Kanamycin/hygromycin resistant primary transformants (T0 generation) with established root systems were transferred to soil and allowed to self fertilize.

### Plant growth conditions

Wild-type tobacco plants and T1 progeny resulting from the self-fertilization of transgenic plants were germinated in sterile agar medium containing Murashige and Skoog salts supplemented with 1% (w/v) Suc (plus kanamycin 100mg for the transformants) and grown to seed in soil (Levington F2, Fisons, Ipswich, UK) and lines of interest were identified by Western blot and qPCR. Wild-type plants used in this study were a combined group of WT and null segregants from the ictB over-expressing lines verified by PCR. Comparative analysis of these groups can be seen in Supplementary Fig. S2 at *JXB* online. For experimental study, T2 progeny seeds were germinated on soil in controlled environment chambers at an irradiance of 130 μmol photons m^–2^ s^–1^, 22 °C, a relative humidity of 60%, in a 12h photoperiod. Plants were transferred to individual 8cm pots and grown for 2 weeks at 130 μmol photons m^–2^ s^–1^, 22 °C, a relative humidity of 60%, in a 12h photoperiod. Plants were transferred to larger pots (17cm across and 23cm deep) and cultivated in a controlled environment greenhouse (16h photoperiod, 25–30/20 °C day/night, and natural light supplemented with high-pressure sodium light bulbs, giving between 200–350 μmol m^–2^ s^–1^ (low light), 600–1 400 μmol m^–2^ s^–1^ (high light) from the pot level to the top of the plant, respectively). Positions of the plants were changed daily and watered with a nutrient medium (Hoagland and Arnon, 1950). Four leaf discs (0.8cm diameter), for the analysis of SBPase and FBPA activities were taken from the same areas of the leaf used for photosynthetic measurements, immediately plunged into liquid N_2_ and stored at –80 °C. Leaf areas were calculated using standard photography and ImageJ software (imagej.nih.gov/ij).

### Protein extraction and Western blotting

Leaf discs sampled as described above were ground in liquid nitrogen and protein quantification determined (Harrison *et al.*, 1998). Samples were loaded on an equal protein basis, separated using 12% (w/v) SDS-PAGE, transferred to polyvinylidene difluoride membrane, and probed using antibodies raised against SBPase and FBPA. Proteins were detected using horseradish peroxidase conjugated to the secondary antibody and the ECL chemiluminescence detection reagent (Amersham, Buckinghamshire, UK). SBPase antibodies are previously characterized in Lefebvre *et al.* (2005) and FBPA antibodies were raised against a peptide from a conserved region of the protein [C]-ASIGLENTEANRQAYR-amide, Cambridge Research Biochemicals, Cleveland, UK.

### Determination of SBPase activity by phosphate release

SBPase activity was determined by phosphate release as described previously (Lefebvre *et al.*, 2005). Immediately after photosynthesis measurement, leaf discs were isolated from the same leaves and frozen in liquid nitrogen. For analysis, leaf discs were ground to a fine powder in liquid nitrogen in extraction buffer (50mM HEPES, pH 8.2; 5mM MgCl; 1mM EDTA; 1mM EGTA; 10% glycerol; 0.1% Triton X-100; 2mM benzamidine; 2mM aminocapronic acid; 0.5mM phenylmethylsulphonylfluoride; 10mM dithiothreitol) and the resulting solution centrifuged for 1min at 14 000×*g* at 4 °C. The resulting supernatant (1ml) was desalted through an NAP-10 column (Amersham) and the eluate aliquoted and stored in liquid nitrogen. For the assay, the reaction was started by adding 20 μl of extract to 80 μl of assay buffer (50mM TRIS, pH 8.2; 15mM MgCl_2_; 1.5mM EDTA; 10mM dithiothreitol; 2mM SBP) and incubated at 25 °C for 30min. The reaction was stopped by the addition of 50 μl of 1M perchloric acid and centrifuged for 10min at 14 000×*g* at 4 °C. Samples (30 μl) and standards (30 μl, PO^3-^_4_ 0.125–4 nmol) in triplicate were incubated for 30min at room temperature following the addition of 300 μl of Biomol Green (Affiniti Research Products, Exeter, UK) and the *A*_620_ was measured using a microplate reader (VERSAmax, Molecular Devices, Sunnyvale, CA).

### Determination of FBPA activity

Desalted protein extracts were evaluated for FBPA activity as described previously by Haake *et al.* (1998).

### cDNA generation and quantitative RT-PCR

Total RNA was extracted from tobacco leaf samples using the NucleoSpin^®^ RNA Plant Kit (Macherey-Nagel, Fisher Scientific, UK). cDNA was synthesized using 1 μg total RNA in 20 μl using the oligo-dT primer according to the protocol in the RevertAid Reverse Transcriptase kit (Fermentas, Life Sciences, UK).

The PCR reaction contained 10mM of each primer, 1.3× *Taq* polymerase buffer, 0.30mM dNTPs, 1.5 units of *Taq* polymerase (BRL), and 2 μl of RT reaction mixture (100ng of RNA) in a total volume of 25 μl. The final concentration was 4ng μl^–1^ of reaction mixture. The amplification reactions included 26 cycles of 30 s at 94 °C, 15 s at 60 °C, and 15 s at 72 °C. PCR products were fractionated on 1.5% agarose gel. Primers ictBf: AAGACAGCAGCAACAACTTC; NOSr: TGCCAAATGTTTGAACGATCG were used to amplify the transgene.

### Chlorophyll fluorescence imaging

Chlorophyll fluorescence measurements were performed on 3-week-old tobacco seedlings that had been grown in a controlled environment chamber at 130 μmol mol^–2^ s^–1^ and ambient (400 μmol mol^–1^) CO_2_. Three days prior to chlorophyll fluorescence imaging, plants were transferred to the greenhouse and grown in natural irradiance with supplementary light to maintain the levels between 400–600 μmol m^–2^ s^–1^ PPFD at bench level. Chlorophyll fluorescence parameters were obtained using a chlorophyll fluorescence (CF) imaging system (Technologica, Colchester, UK; Barbagallo *et al.*, 2003; Baker and Rosenqvist, 2004). The operating efficiency of photosystem II (PSII) photochemistry, *F*_q_′/*F*_m_′, was calculated from measurements of steady-state fluorescence in the light (*F*′) and maximum fluorescence in the light (*F*_m_′) was obtained after a saturating 800ms pulse of 5 500 μmol m^–2^ s^–1^ PPFD using the following equation *F*_q_′/*F*_m_′ = (*F*_m_′-*F*′)/*F*_m_′. Images of *F*_q_′/*F*_m_′ were taken under stable PPFD of 400 and 800 μmol m^–2^ s^–1^ PPFD (Oxborough and Baker, 1997; Baker *et al.*, 2001). Measurements on tobacco seedlings were performed on 3-week-old plants grown in a controlled environment chamber with 130 μmol m^–2^ s^–1^ PPFD and ambient 400 μmol mol^–1^ CO_2_.

### Gas exchange measurements

The response of net photosynthesis (*A*) to intracellular CO_2_ concentration (*C*_i_) was measured using a portable gas exchange system (LI-COR 6400; LI-COR, Lincoln, NE). Illumination at 2000 μmol m^–2^ s^–1^ was provided by a red–blue light source attached to the leaf curve. Measurements of *A* were made at ambient CO_2_ concentration (*C*_a_) of 400 μmol mol^–1^, before *C*_a_ was decreased step-wise to the lowest concentration of 50 μmol mol^–1^ and then increased step-wise to an upper concentration of 2000 μmol mol^–1^. Leaf temperature and vapour pressure deficit (VPD) were maintained at 25 °C and 1±0.2 kPa, respectively. To calculate the maximum saturated CO_2_ assimilation rate (*A*_max_), maximum carboxylation rate (*Vc*_max_) and maximum electron transport flow (*J*_max_), the C3 photosynthesis model (Farquhar *et al.*, 1980), was fitted to the *A*/*C*_i_ data using a spreadsheet provided by Sharkey *et al.* (2007).

### Diurnal photosynthesis

The diurnal response of leaf photosynthesis (*A*) and stomatal conductance (*g*_s_) of a young expanding leaf (15–16cm in length) were measured every 2h between 06.30h and 20.00h. Measurements were made using a portable gas exchange system (LI-COR 6400). Light levels at each time point were set at the ambient light over the day: light levels ranged from 0 μmol m^–2^ s^–1^ to 350 μmol m^–2^ s^–1^, at mid-plant levels on the day of analysis. Measurements of *A* and *g*_s_ were recorded at steady-state (*c*. 2min) and used to calculate intrinsic water-use efficiency (*A*/*g*_s_=WUE_i_). To estimate the integrated carbon gain (*A*’) and water loss (*g*_s_’) over the measurement period, the area under the diurnal curved was calculated for each transgenic line.

### Statistical analysis

All statistical analyses were done by comparing ANOVA, using Sys-stat, University of Essex, UK. The differences between means were tested using the Post hoc Tukey test (SPSS, Chicago).

## Results

### Production and selection of tobacco transformants

The full-length *Arabidopsis thaliana* (*Arabidopsis*) FBPA cDNA (At4g38970) and the putative-inorganic carbon transporter B (*ictB*) coding sequences (YP399376) (linked to the *Brachypodium distachyon* SBPase transit peptide (XP_003564625) were used to generate three cover-expression constructs driven by the CaMV 35S or FMV promoter; B2-FBPA, B2-ictB, and FB-TB in the vector pGWB2 (see Supplementary Fig. S1 at *JXB* online). Following leaf disc transformation of previously generated SBPase (At3g55800) over-expressing (lines 30 and 60) (Lefebvre *et al.*, 2005) or wild-type (WT) tobacco plants. Primary transformants (57) (T0 generation) were rooted on hygromycin+kanamycin-containing medium, subsequently transferred to soil, and grown until maturity. Plants expressing the integrated transgenes were screened using RT-PCR (data not shown).

**Fig. 1.**
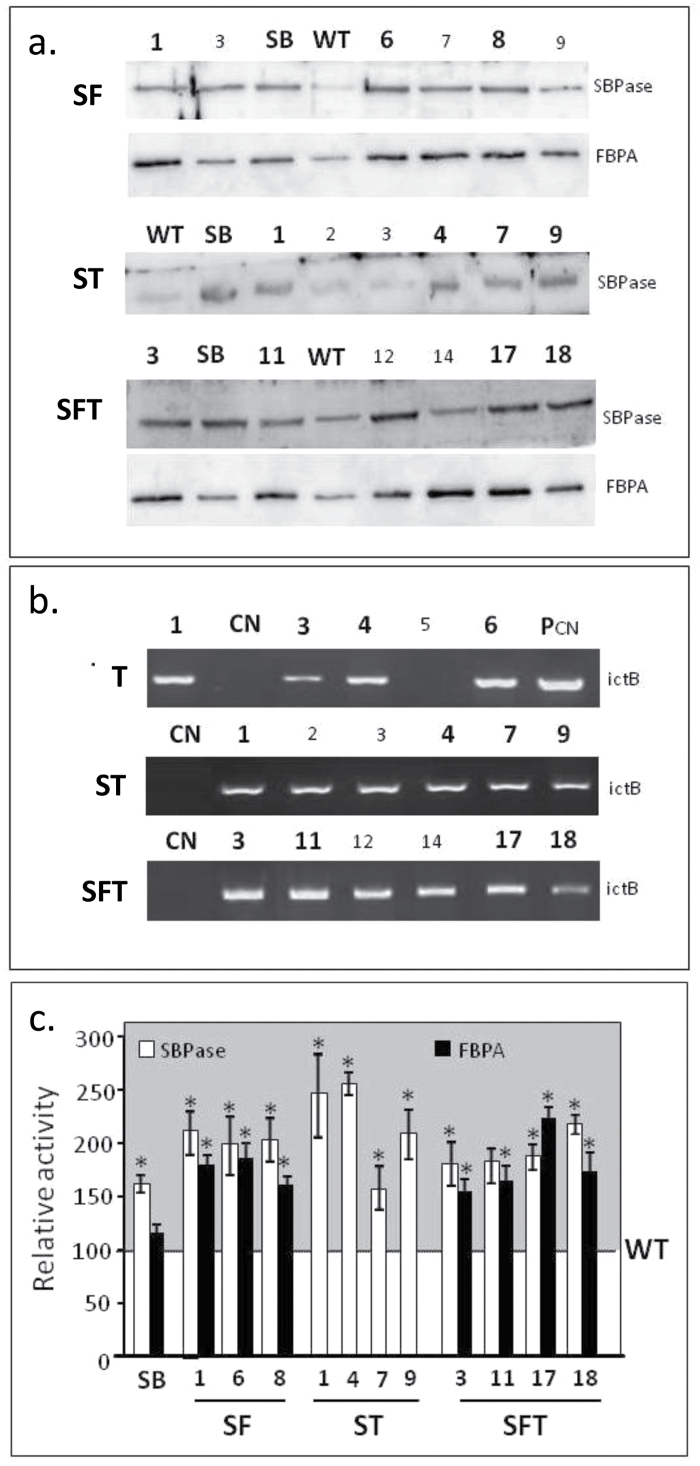
Molecular and biochemical analysis of the transgenic plants over-expressing SBPase (S), FBPA (F), and ictB (T). (a) Immunoblot analysis (SBPase and FBPA) of protein extracts from the fully expanded leaves of evaluated lines. (b) ictB transcript levels in lines T, ST, and SFT compared with a non-transformed control (CN); P_CN_=plasmid control. (c) SBPase and FBPA enzyme activity in fully expanded leaves from SF, ST, and SFT lines used in this study compared with WT and S lines previously studied (Lefebvre *et al.*, 2005). Enzyme assays represent data from four or five independent plants per line (see Supplementary Fig. S3 at *JXB* online). An asterisk indicates lines which are statistically different from WT and SB lines (*P <0.05). Numbers in bold represent lines used in this study.

Western-blot analysis of T1 progeny from six selected lines (based on RT-PCR data) for transgenic tobacco expressing ictB (T), SBPase+FBPA (SF), SBPase+ictB (ST), and SBPase+FBPA+ictB (SFT) were carried out using WT and SBPase over-expressing lines (S) as a control. Western-blot analysis revealed a number of plants over-expressing SBPase for lines SF, ST, and SFT and plants over-expressing FBPA in lines SF and SFT (Fig. 1a). No significant difference in Rubisco protein levels was observed between WT and the S and transgenic groups (see Supplementary Fig. S3a at *JXB* online).

Due to the difficulty in generating an antibody for ictB, semi-quantitative RT-PCR was used to detect the presence of the transcript in the ictB-expressing plant lines, T, ST, and SFT (Fig. 1b) and, while no transcript was detected in non transformed controls, transcript accumulation was easily observed in all transgenic lines selected for study.

In addition to Western-blot analysis carried out on T1 plants, total extractable SBPase and FBPA activity were analysed in newly fully expanded leaves of the T2 progeny used for the experimental determination of chlorophyll fluorescence and photosynthetic parameters. This analysis showed that these plants had increased levels of both SBPase and FBPA activity (Fig. 1c); SBPase activities ranged from 130–280% and FBPA activities in lines co-expressing FBPA, in addition to SBPase, displayed an increase of between 140% and 250%. The full set of assays showing the variation between plants for both SBPase and FBPA activities can be seen in Supplementary Fig. S3b, c at *JXB* online.

### Chlorophyll fluorescence imaging reveals increased photosynthetic efficiency in young transgenic seedlings

**Fig. 2.**
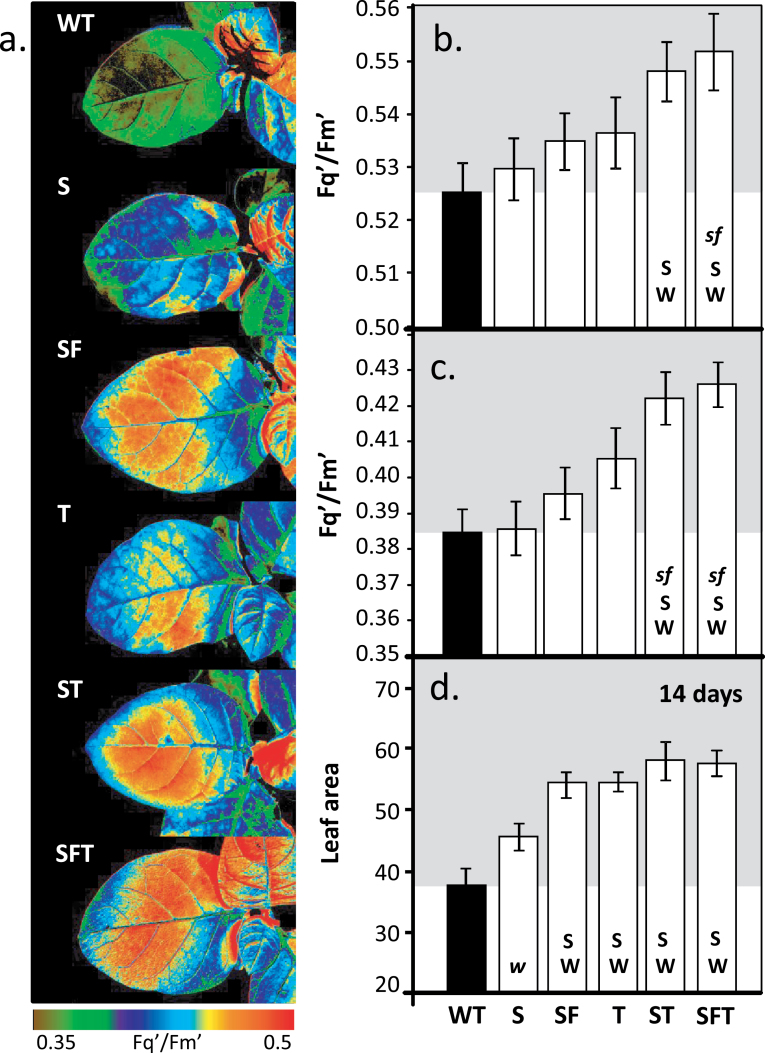
Determination of photosynthetic capacity and leaf area in transgenic seedlings using fluorescence imaging. WT and transgenic plants were grown in controlled environment conditions with a light intensity of 130 μmol m^–2^ s^–1^, a 12/12h light/dark cycle for 14 d, and chlorophyll fluorescence was used to determine *F*_q_′/*F*_m_′ (maximum PSII operating efficiency) at two light intensities: (a) 800, (b) 400, and (c) 800 μmol m^–2^ s^–1^. (d) Leaf area at time of analysis. Scale bar represents an *F*_q_′/*F*_m_′ of 0.35–0.50. The data were obtained using 12–16 individual plants from 3–4 independent transgenic lines. Significant differences (<0.05) between lines is shown by capital letters [i.e. SFT lines are significantly bigger than wild-type (W) and SBPase over-expressing lines (S)]. Lower case italic lettering indicates lines that are just below significance (>0.05– <0.062). Lines over-expressing SBPase (S), SBPase and FBPA (SF), ictB (T), SBPase and ictB (ST), and SBPase, FBPA, and ictB (SFT) are represented.

In order to screen for potential changes in photosynthesis in 14-d-old seedlings (T2 progeny), chlorophyll *a* fluorescence imaging was used to examine the quantum efficiency of PSII photochemistry (*F*_q_′/*F*_m_′) (Baker, 2008; Murchie and Lawson, 2013). Analysis of plants over-expressing SBPase alone did not show an increase in *F*_q_′/*F*_m_′. However, plants over-expressing FBPA and/or ictB in conjunction with SBPase (ST and SFT) had a significantly higher *F*_q_′/*F*_m_′ at an irradiance of 400 μmol m^–2^ s^–1^ when compared with either WT or SBPase over-expressing plants (Fig. 2a, b). At a higher light level (800 μmol m^–2^ s^–1^) both the ST and SFT plants had a significantly higher *F*_q_′/*F*_m_′ compared with WT, S, and SF lines (Fig. 2c). From images taken during the fluorescence analysis of the seedlings, it was shown that the leaf area for SF, T, ST, and SFT plants was significantly larger than both WT and S (Fig. 2d). Differences in leaf area were also apparent in 8-d-old seedlings but, at this stage, no significant difference was observed between WT and S plants (see Supplementary Fig. S4 at *JXB* online).

### Photosynthetic CO_2_ assimilation rates are increased in mature plants grown in high light in the greenhouse

**Fig. 3.**
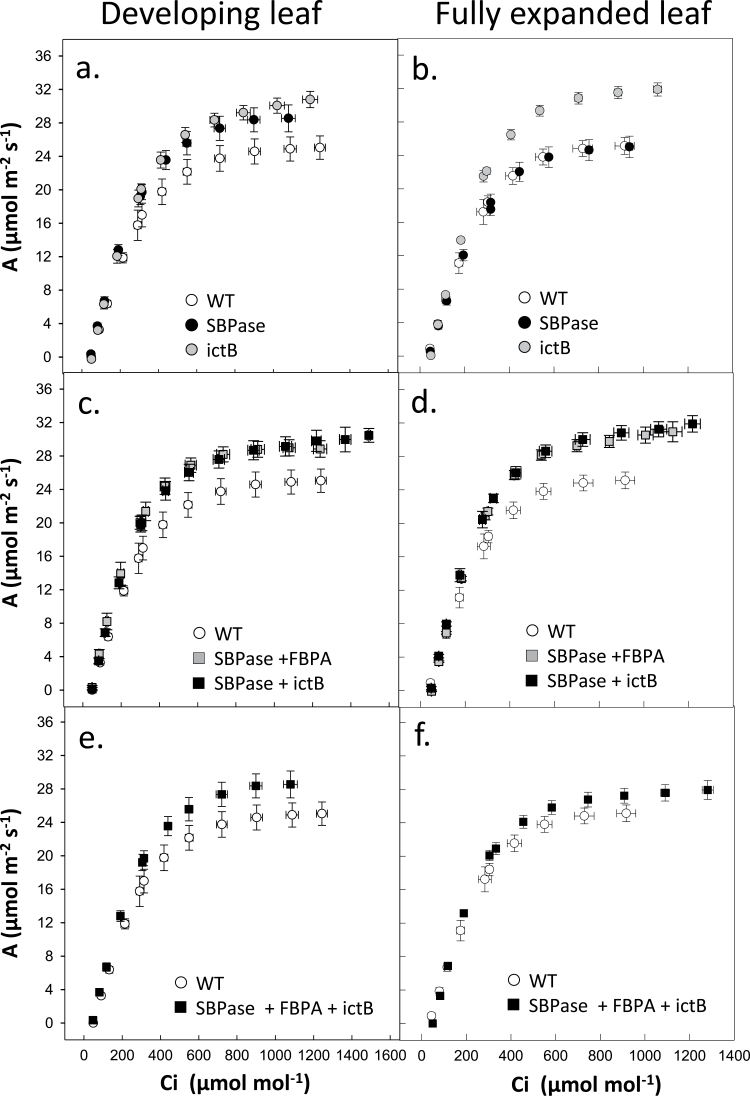
Photosynthetic responses of WT and transgenic plants grown in high light in the greenhouse. Photosynthetic carbon fixation rates were determined as a function of increasing CO_2_ concentrations (*A*/*C*_i_) at saturating-light levels in developing leaves (11–13cm in length) and fully expanded (leaf 8) from WT and transgenic plants. (a, c, e) Developing leaves; (b, d, f) fully expanded leaves. Plants were grown in natural light conditions in the greenhouse, light levels were between 600 and 1500 μmol m^–2^ s^–1^ (supplemental light maintain a minimum of 600 μmol m^–2^ s^–1^). White circles, WT plants (*n*=6); black circles, SBPase over-expressing plants (*n*=8) (Lefebvre *et al.*, 2005). Values are from 12–16 individual plants from 3–4 independent transgenic lines.

**Fig. 4.**
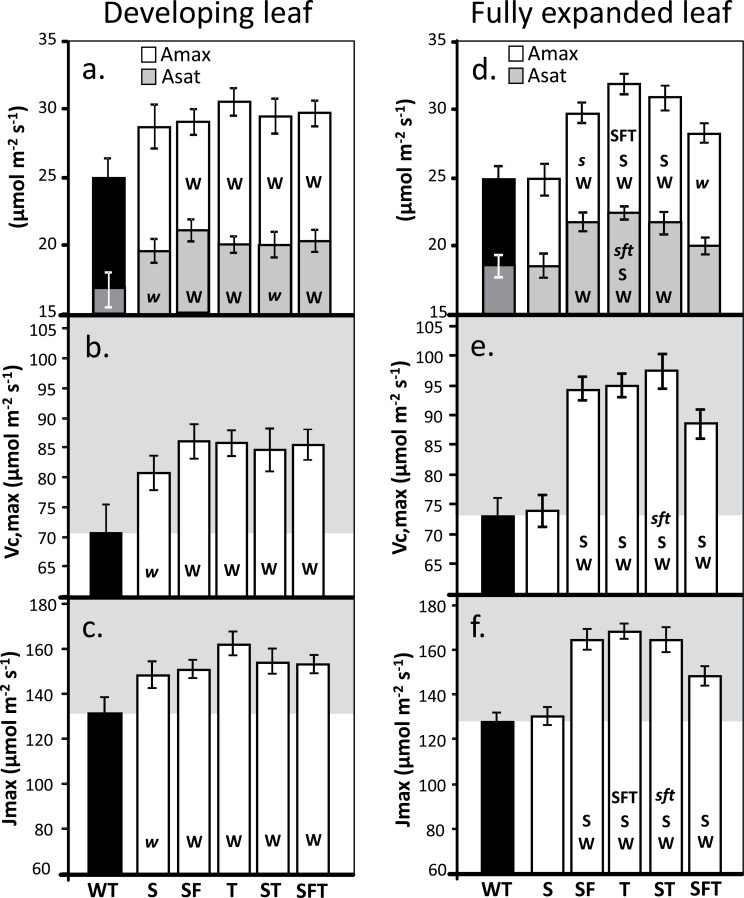
Rubisco activity and *J*_max_ derived from *A*/*C*_i_ response curves. These data are derived from the *A*/*C*_i_ response curves shown in Fig. 3 using the equations published by von Caemmerer and Farquhar (1981). Developing leaves (12cm leaves) and fully expanded leaves are shown. Values represent four plants from 3–4 individual lines (12–16 plants) for each transgene set. Lines over-expressing SBPase (S), SBPase and FBPA (SF), ictB (T), SBPase and ictB (ST), and SBPase, FBPA, and ictB (SFT) are represented. Significant differences (<0.05) are represented by capital letters. Lower case italic lettering indicates lines that are just below significance (>0.05 and <0.1).

Following chorophyll fluorescence analysis, plants were moved into large pots and grown for a further 4 weeks in the greenhouse, in natural light with supplementation providing light levels fluctuating between 600–1400 μmol m^–2^ s^–1^. The rate of CO_2_ assimilation (*A*) was determined as a function of internal CO_2_ concentration (*C*_i_) in both the newest fully expanded leaf and in young expanding leaves (Fig. 3). In all the transgenic plants analysed in this study, the rate of *A* in developing leaves was significantly greater than that in WT plants at *C*_i_ concentrations above *c*. 300 μmol mol^–1^ (Fig. 3). This was accompanied by a significantly greater light-saturated rate of photosynthesis (*A*_sat_) in all transgenic plants compared with the WT control (Fig. 4a). Further analysis of the *A*/*C*_i_ curves illustrated that the light- and CO_2_-saturated rate of photosynthesis (*A*_max_) was also significantly greater in all transgenic plants, with the exception of the plants over expressing of SBPase alone (Fig 4a, b). However, no significant enhancements of *A*_sat_ or *A*_max_ were observed between the different transgenic plants. Similarly the maximum rate of Rubisco carboxylation (*Vc*_max_) and electron transport (*J*_max_), calculated from the *A*/*C*_i_ curves, were statistically greater in all transgenic plants compared with the wild type in developing leaves, but again no significant differences between the transgenic lines was observed (Fig. 4b, c).

In the fully expanded leaves of the same plants, the differences observed were more complex. No significant differences in *A*_sat_ or *A*_max_ were observed in plants over expressing SBPase alone compared with the WT (Figs 3, 4d). By contrast, plants over-expressing both SBPase and FBPA or SBPase and ictB or SBPase, FBPA, and ictB were all shown to have higher *A*_max_ than the WT (Fig. 4b). On the other hand, at ambient CO_2_ in saturating light (*A*_sat_), plants over-expressing SBPase, FBPA, and ictB showed no significant difference in *A*_sat_ compared with the WT, however, the double expressing SF and ST had a significantly higher *A*_sat_ than the WT. Transgenic tobacco over-expressing T alone showed significantly higher *A*_max_ values compared with the triple over-expressing (SFT) plants, and SBPase alone, whilst the two double over-expressing lines were both significantly greater than plants over-expressing SBPase alone (Fig. 4d). *Vc*_max_ and *J*_max_ were statistically greater in all transgenic plants with the exception of those over-expressing SBPase alone, compared with the wild type and the single over-expressing SBPase plants (Fig. 4e, f). Furthermore, plants over-expressing itcB alone had a significantly high *J*_max_ when compared with the triple over-expressing (SFT) plants (Fig. 4f).

**Fig. 5.**
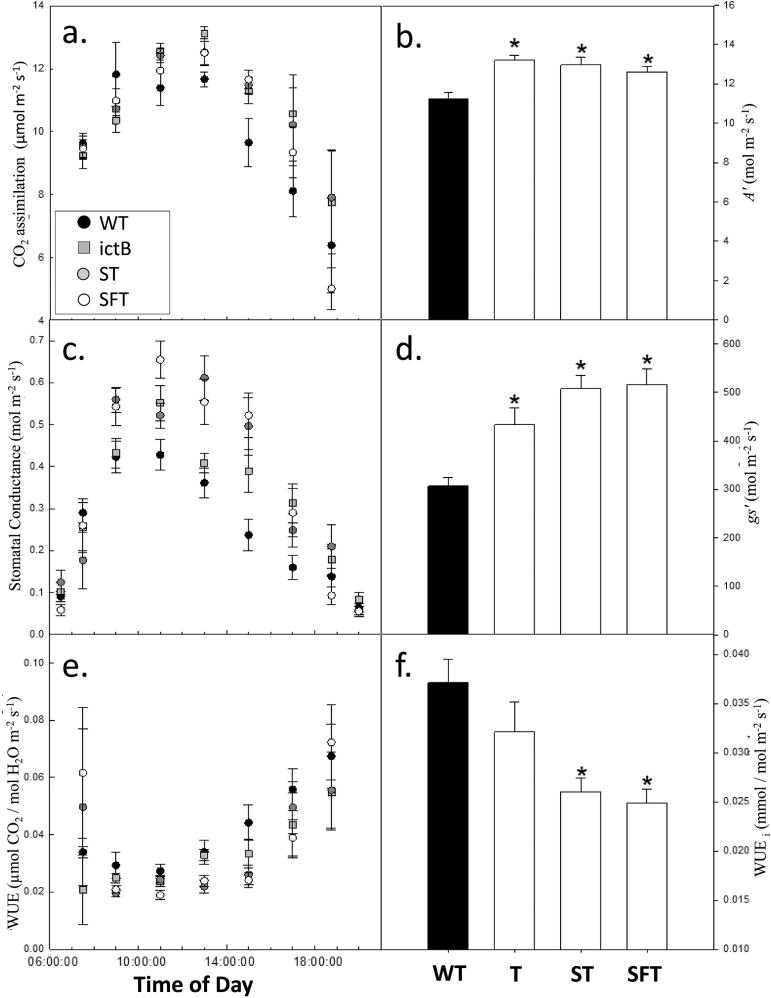
The diurnal progression of CO_2_ assimilation, stomatal conductance, and water use efficiency in the WT and transgenic plants. Plants were grown in greenhouse conditions as in Fig. 3. (a) Leaf photosynthesis (*A*), (c) stomatal conductance, and (e) intrinsic water use efficiency (*A*/*g*_s_=WUE_i_) were monitored over a 14h period. Each symbol represents the mean ±SE of five plants. For each transgenic group, two plants from each of four independent transgenic lines were evaluated and grouped together (eight plants per group). The integrated (b) carbon gain (*A*’), (d) water loss (g_s_’), and (f) total water use efficiency were calculated. An asterisk denotes a significant (<0.05) difference compared with WT plants. Lines over-expressing ictB (T), SBPase and ictB (ST), and SBPase, FBPA, and ictB (SFT) are represented.

To investigate further the *in situ* response of photosynthesis to the over-expression of ictB combined with increased enzyme activities (SBPase/FBPA), instantaneous measurements of *A*, *g*_s_, and intrinsic water use efficiency (WUE_i_) were measured in young expanding leaves of the wild type and transgenic lines. Lines over-expressing ictB (T), SBPase+ictB (ST) or SBPase+FBPA+ictB displayed an increase in photosynthetic rate of 19%, 16%, and 12%, respectively (based on measurements obtained between 09.00h and 19.00h) (Fig. 5a, b). These increases in *A* were accompanied by increased *g*_s_ (Fig. 5c, d) which resulted in lines over-expressing ictB in conjunction with either SBPase (ST) or SBPase and FBPA (SFT) having a lower water use efficiency, possibly due to a greater stomatal conductivity (Fig. 5e, f). Interestingly no significant decrease in WUE was apparent in plants over-expressing ictB alone compared with the WT.

### Increased SBPase and FBPA activity and expression of ictB stimulates growth in high light

**Fig. 6.**
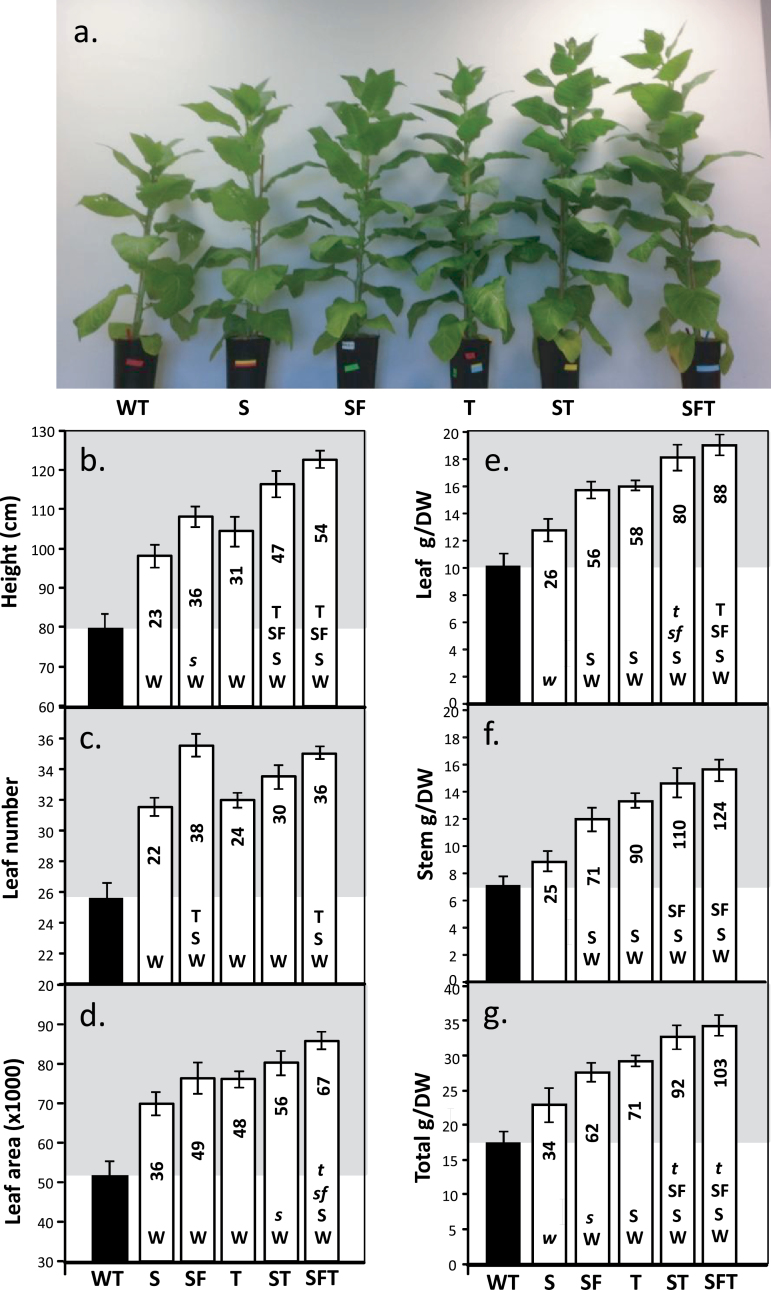
Growth analysis of WT and transgenic plants grown in greenhouse conditions. Plants were grown for 2 weeks at 130 μmol m^–2^ s^–1^ light intensity in long days (12/12h days) before being transferred to the greenhouse and supplemented light (16/8h days), at maximum 600–1400 μmol m^–2^ s^–1^ light intensity for an additional 4 weeks (6 weeks in total). Plants were harvested when the first sign of flower development became apparent. (a) Appearance of plants 6 weeks after planting. Lines over-expressing SBPase (S), SBPase and FBPA (SF), ictB (T), SBPase and ictB (ST), and SBPase, FBPA, and ictB (SFT) are represented. (b) Plant height, (c) leaf number, (d) leaf area, and (e–g) biomass. Results are representative of 4–5 plants from 3–4 individual lines (12–16 plants in total). Significant differences (<0.05) are represented by capital letters indicating if each specific line is significantly different from another. Lower case italic lettering indicates lines that are just below significance (>0.05 and <0.1). The percentage increases over the wild type are indicated.

The group of plants used for photosynthetic analysis described above were destructively harvested after 4 weeks further growth and the height, leaf number, and leaf area determined (Fig. 6). An increase in total biomass was also observed in all transgenic lines and, in the ST and SFT lines, a doubling of the dry weight was observed (Fig. 6g). These increases in total biomass were due to an increase in both leaf and stem dry weights (Fig. 6e, f). For example, plants over-expressing SFT showed an 88% and 124% increase in biomass for leaf and stem material, respectively. The increases in total biomass for the SFT lines (103%) were significantly higher than S (+34%), T (+71%), and SF (+62%) indicating a positive effect of the additional transgenes (Fig. 6). The full set of data for these plants can be found in Supplementary Fig. S5 and Supplementary Table S1at *JXB* online. Similar increases in height and leaf number were also evident in a non-destructive assessment of growth at an earlier stage in development (28 d after planting: see Supplementary Figs S6 and S7 at *JXB* online). The increase in above-ground biomass in these transgenic plants was not at the expense of reduced root growth (see Supplementary Fig. S8 at *JXB* online).

### The impact of manipulation of the C3 cycle on photosynthetic CO_2_ assimilation and biomass when growth was in simulated shade

**Fig. 7.**
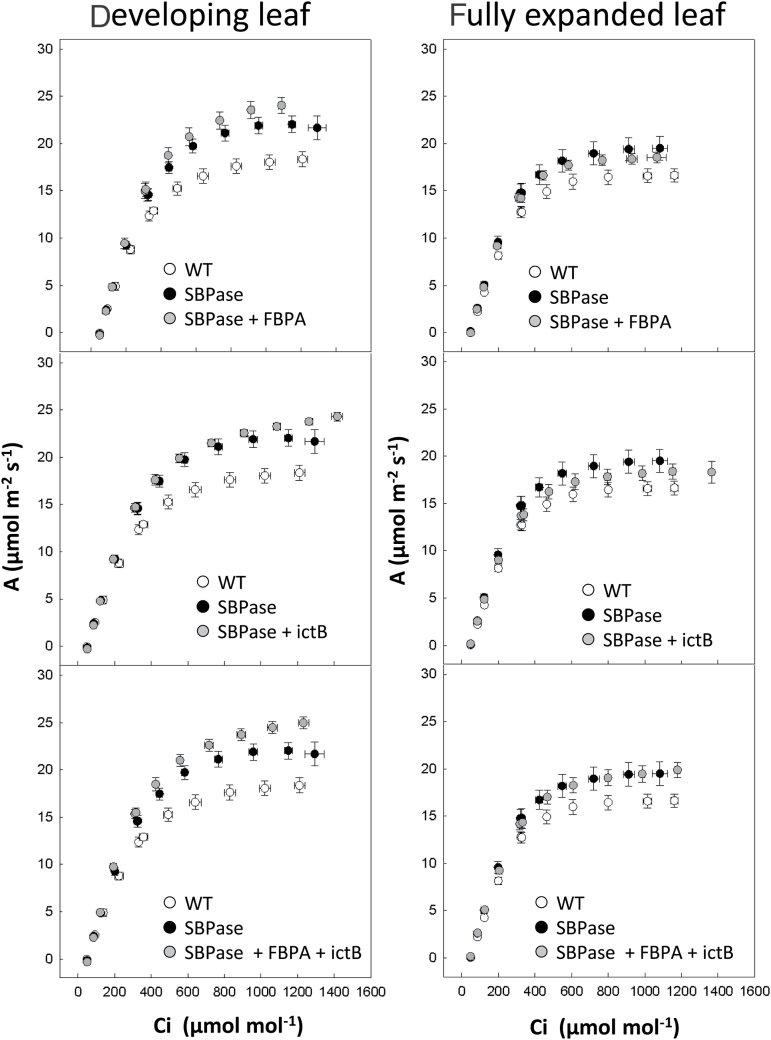
Photosynthetic responses of WT and transgenic plants grown in low light. Photosynthetic capacity of developing leaves (11–13cm) and fully expanded leaves (leaf 7) from low-light, greenhouse-grown wild-type and transgenic plants. Plants were grown in supplemented, fluctuating light at a maximum of 200–350 μmol m^–2^ s^–1^ light intensity. Photosynthetic carbon fixation rates were determined as a function of increasing CO_2_ concentrations at saturating-light levels. White circles represent the results from six wild-type plants, and black circles represent the results from eight SBPase over-expressing lines (Lefebvre *et al.*, 2005). Grey circles represent 10–17 individual plants from 3–4 independent transgenic lines over-expressing SBPase+FBPA, SBPase+ictB, and SBPase, FBPA,+ictB.

**Fig. 8.**
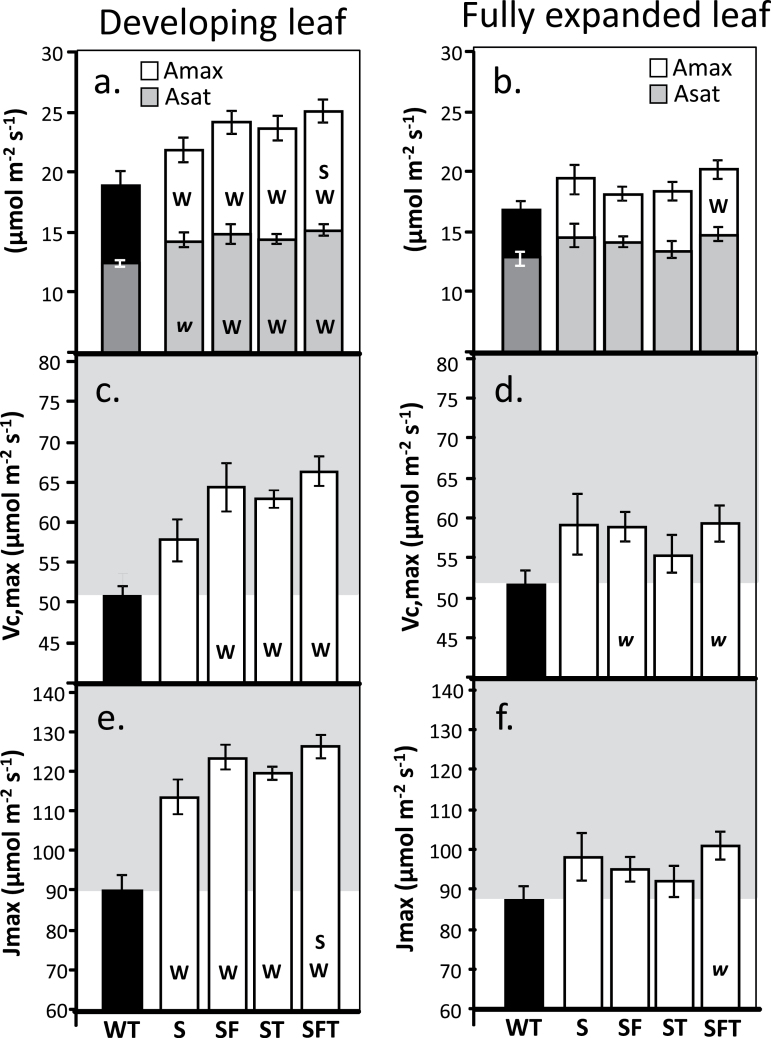
Rubisco activity and *J*_max_ derived from *A*/*C*_i_ response curves. These data are derived from the *A*/*C*_i_ response curves shown in Fig. 8 using the equations published by von Caemmerer and Farquhar (1981). Plants were grown in long days for 2 weeks at 200–350 μmol m^–2^ s^–1^ light intensity followed by 5 weeks in natural light conditions in the greenhouse at 200–350 μmol m^–2^ s^–1^ light intensity. Developing leaves (11–13cm leaves) and fully expanded leaves. Values represent four plants from either 3–4 independent lines (12–16 plants) for each transgene set. Significant differences (<0.05) are represented by capital letters. Lower case italic lettering indicates lines that are just below significance (>0.05 and <0.1).

*A/*C_i_ response curves were determined for plants over-expressing SBPase, SBPase+FBPA, SBPase+ictB, and SBPase+FBPA+ictB grown under simulated shade conditions (natural light, under shading at 200–350 μmol m^–2^ s^–1^ light intensity) (Fig. 7). These analyses revealed that *A*_sat_ and *A*_max_ were increased in developing leaves of transgenic plants compared with the WT plants and *A*_max_ in the SFT plants was significantly greater than for SBPase alone (Fig. 8a). However, with the exception of the SFT plants, little or no difference in either *A*_sat_ or *A*_max_ was observed in fully expanded leaves (Fig. 8b). Similarly, *Vc*_max_ and *J*_max_ were both enhanced in developing leaves of the majority of the transgenic plants compared with the WT (Fig. 8c, e). The SFT plants showed a further significant enhancement of *J*_max_ over SB alone (Fig. 8a, e). By contrast, analysis of *Vc*_max_ and *J*_max_ in fully expanded leaves revealed little significant difference between transgenic and WT plants (Fig. 8d, f).

**Fig. 9.**
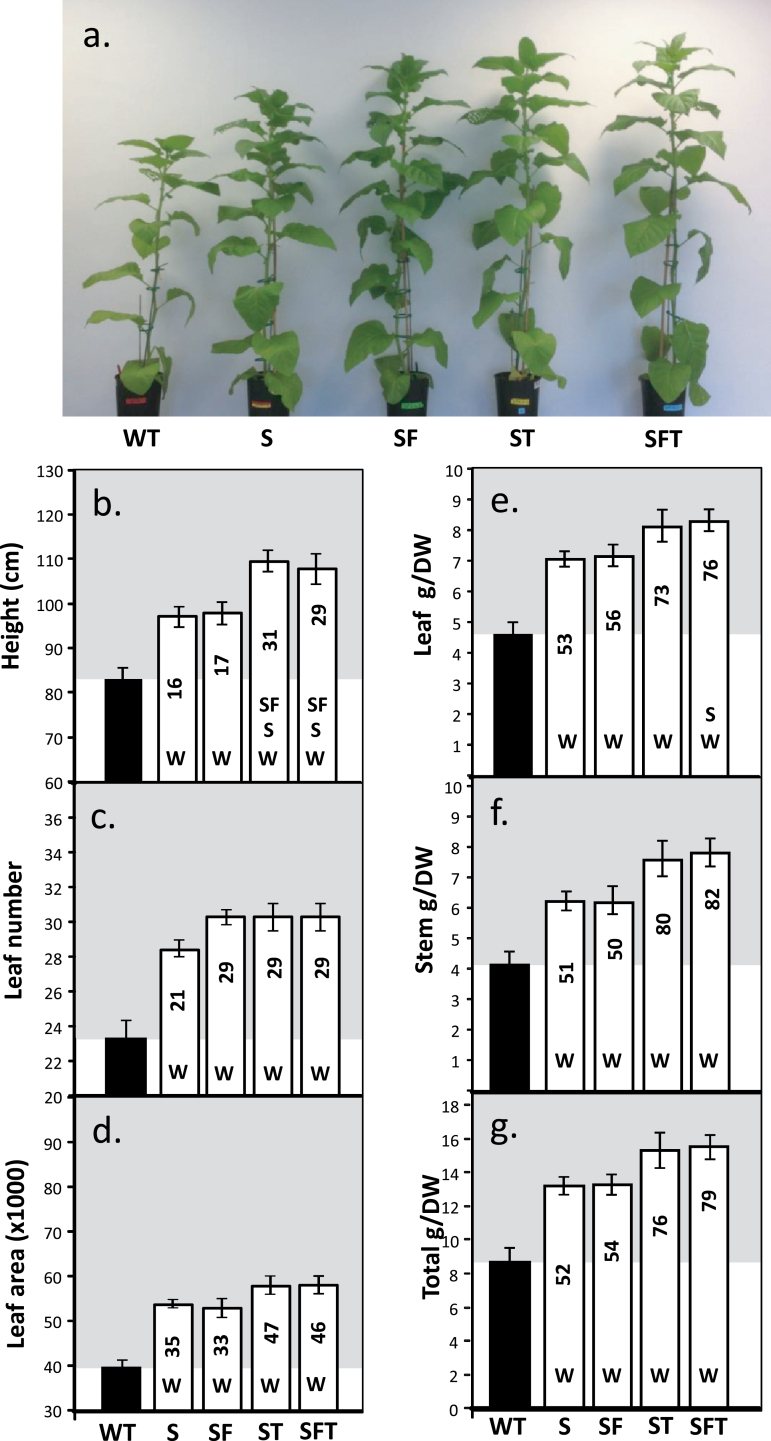
Growth analysis of WT and transgenic plants grown in low light in the greenhouse. Plants were grown for 2 weeks at 130 μmol m^–2^ s^–1^ light intensity in long days (12/12h days) before being transferred to the greenhouse and supplemented light (16/8h days), at a maximum 200–350 μmol m^–2^ s^–1^ light intensity for an additional 5 weeks (7 weeks in total). Plants were harvested when the first sign of flower development became apparent. (a) Appearance of plants after 7 weeks. Lines over-expressing SBPase (S), SBPase and FBPA (SF), ictB (T), SBPase and ictB (ST), and SBPase, FBPA, and ictB (SFT) are represented. (b) Plant height, (c) leaf number, (d) leaf area, and (e–g) biomass. Results are representative of 4–5 plants from 3–4 individual lines (12–16 plants in total). Significant differences (<0.05) are represented by capital letters indicating if each specific line is significantly different from another. Lower case italic lettering indicates lines that are just below significance (>0.05 and <0.1). The percentage increases over the wild type are indicated.

Significant differences in growth and biomass were also clearly evident in the transgenic plants grown in these simulated shade conditions (Fig. 9). This analysis revealed that height, leaf number, and leaf area were increased consistently in transgenic plants when compared with WT plants (Fig. 9). Total dry weight was increased by between 52% and 79% for lines S, SF, ST, and SF. The full set of data for low-light-grown plants can be seen in Supplementary Fig. S9 and Supplementary Table S1 at *JXB* online.

## Discussion

The products of photosynthesis are the primary determinant of plant productivity and improving photosynthetic efficiency has been widely proposed as a key target for improving crop yield (Long *et al.*, 2006; Zhu *et al.*, 2010; Parry *et al.*, 2011; Raines, 2011). It has been shown previously that increasing the activity of two enzymes of the C3 cycle, SBPase (Lefebvre *et al.*, 2005) and FBPA (Uematsu *et al.*, 2012) in transgenic tobacco resulted in an increase in photosynthetic carbon assimilation and growth. Furthermore, the expression of the inorganic CO_2_ transporter B (ictB) in transgenic tobacco (Lieman-Hurwitz *et al.*, 2003) also resulted in an increase in photosynthesis and biomass. These studies showed that the stimulation of photosynthesis was not seen under all experimental conditions, highlighting the need to consider different strategies to ensure success of this approach in a range of environmental conditions. These results, together with the analysis of the C3 cycle using a modelling approach (Poolman *et al.*, 2000: Zhu *et al.*, 2007), suggested that a re-engineering of multiple enzymes of the C3 cycle had the potential to achieve the gains in photosynthesis and growth required to provide sufficient food in the 21st century. In this study, it has been shown that increasing the levels of SBPase, FBPA, and ictB simultaneously in the same plant, compared with SBPase, FBPA or ictB alone or in pairs, resulted in a substantial and significant impact on photosynthesis and the biomass yield of tobacco grown in greenhouse conditions.

The plants in this study were grown in conditions as close to natural as possible; the pots containing the plants were grown in the greenhouse with natural lighting, plants were positioned such that, at maturity, a near-to-closed canopy was achieved and the temperature range was similar to the ambient external environment. Previously, it was shown that over-expression of FBPA in tobacco resulted in a stimulation of photosynthesis and growth when plants were grown under a square wave lighting environment and elevated CO_2_ conditions. However, the effect of FBPA over-expression was much less in plants grown in ambient CO_2_ conditions (Uematsu *et al.*, 2012). Interestingly, in this current study, it is shown that, even in ambient CO_2_, over-expression of FBPA together with SBPase has a positive effect on growth in high light when compared with either WT plants or plants over-expressing SBPase alone. However, the cumulative effect of SBPase plus FBPA co-expression was not observed when plants were grown in low light under simulated shading. The over-expression of FBPA and SBPase would be expected to increase the capacity for regeneration of the CO_2_ acceptor molecule RuBP and this was evident from analysis of the *A*/*C*_i_ response curves from the transgenic lines over-expressing these enzymes. Interestingly, in all of the lines in which *J*_max_ was increased, a stimulation in *Vc*_max_ was also observed, indicating an increase in the carboxylation rate of Rubisco. This may not be surprising as a stimulation in RuBP regeneration will increase the concentration of RuBP, thereby increasing the rate of carboxylation (Wullschleger, 1993; Price *et al.*, 1998; Harrison *et al.*, 2001). Rubisco activation state and RuBP levels were also shown to be higher in transgenic plants where photosynthetic capacity has been increased by the over-expression of SBPase (Lefebvre *et al.*, 2005) or a bifunctional cyanobacterial FBPase/SBPase (Miyagawa *et al.*, 2001).

It has been shown previously that the introduction of ictB into tobacco or *Arabidopsis* results in increased rates of Rubsico-limited photosynthesis together with increased growth [evident under low relative humidity (30%)]—this growth enhancement was, however, not observed under high relative humidity (70%). It is also shown here that, in high light, ictB (under 60% relative humidity) has the ability to increase photosynthesis and growth either when expressed on its own or together with SBPase or SBPase with FBPA. Although the additive effect of ictB with either SBPase or SBPase plus FBPA, was evident in the biomass gain, no enhancement of photosynthesis was detected in the mature leaves of these plants. The function of the *ictB* gene product has not been elucidated, but it has been proposed to be involved in accumulation of inorganic carbon in the cyanobacterium *Synechococcus* (PCC 7942), based on the isolation of a high CO_2_-requiring mutant strain (Bonfil *et al.*, 1998; Kaplan and Reinfold, 1999). However, there was no evidence, from analysis of the *A*/*C*_i_ curve, to support the suggestion that increased photosynthetic rates in the ictB over-expressing plants was only due to a stimulation of the carboxylation reaction of Rubisco. The *A*/*Ci* curves were similar to those observed in transgenic plants with altered FBPA and SBPase levels that showed increased values of *A*_sat_ and *J*_max_. Measurements of photosynthesis revealed that the ictB plants had similar rates of photosynthesis over the diurnal period as did SBPase+ictB or ictB together with both FBPA and SBPase. Interestingly, in all the transgenic plants, the greatest increases in photosynthesis were observed after midday, when the stomatal conductance of WT plants was reduced. It is not clear if these differences were due to a direct effect on stomatal aperture brought about by changes in guard cell C3 enzymes (Lawson *et al.*, 2008, 2014; Lawson, 2009). However, the reduced stomatal conductance in WT plants did not appear to restrict photosynthesis as *C*_i_ values were always greater than *c*. 270 μmol mol^–1^. In the ictB plants, gains in photosynthetic carbon assimilation did not correspond to a significant decrease in WUE_i_. Previous studies on antisense tobacco plants with targeted reduction in photosynthetic enzymes have reported no differences in *g*_s_ between WT and transgenic plants despite large decreases in assimilation rate (Quick *et al.*, 1991*a*, *b*; Hudson *et al.*, 1992; Price *et al.*, 1995) leading to significant reductions in WUE. Although the above studies were not specifically designed to explore stomatal behaviour in these transgenic plants, subsequent research has confirmed that stomata in transgenic plants with impaired carboxylation (von Caemmerer *et al.*, 2004), electron transport (Baroli *et al.*, 2008) or RuBP regeneration (Lawson *et al.*, 2008) can achieve equivalent or even greater *g*_s_ than WT plants and, therefore, have lower WUEs than the WT. The increase in *g*_s_ in these plants was not due to a lowering of *C*_i_, which remained >300 μmol mol^–1^ throughout the measurement period. Therefore, further work is needed to explore the mechanism(s) that link stomatal behaviour with mesophyll demands for carbon assimilation (Lawson, 2009; Lawson *et al.*, 2014; Lawson and Blatt, 2014) particularly in those plants with alteration to *A*.

Simulated shading was also used to explore further the potential of our strategy to increase photosynthesis and yield in plants grown in suboptimal light conditions. Theoretical considerations would predict that, under low CO_2_ and high light, Rubisco activity would limit CO_2_ fixation in C3 plants. In low-light conditions, all of the transgenic lines showed a significant increase in photosynthetic CO_2_ assimilation when compared with the wild type. With only one exception (SFT *A*_max,_ *J*_max_), no significant difference was observed between transgenic lines expressing additional transcripts when compared with SBPase over-expressing lines. These data indicate that, in low-light conditions, light limitation impacts negatively on the benefits seen in photosynthesis observed in the plants expressing multiple transgenes grown in high light. Furthermore, these data imply that the photosynthetic efficiency of young expanding leaves may have a greater impact on plant development than the fully expanded leaves when plants are grown in low-light conditions. It is interesting that greater rates of photosynthesis in the transgenic plants were generally observed in fully expanded leaves in high-light-grown plants whilst, in plants grown under low-light conditions, the fully expanded leaves show limited increases in photosynthesis. These finding are consistent with earlier studies that showed that the main stimulatory effects of increased levels of SBPase occurred earlier in development (Lefebvre *et al.*, 2005) and may also demonstrate the different limitations imposed on photosynthesis between developing and fully expanded leaves (Ölçer *et al.*, 2001).

Chlorophyll fluorescence imaging, used to analyse plants at the seedling stage of development, demonstrated that the positive effect of multigene manipulation on the C3 cycle is evident early in development. The expression of SBPase, FBPA, and ictB in the same plant led to a further increase (11%) in *F*_q_′/*F*_m_′ compared with the increase over WT observed in plants expressing SBPase and FBPA together (2.8%) or plants expressing ictB alone (5.3%). These increases in photosynthetic rates translated into an increase in biomass in all the transgenic lines and, similar to previous studies, SBPase over-expressing lines showed an increase of ~25% in both leaf and stem biomass when grown in high light (Lefebvre *et al.*, 2005). Furthermore, increases in biomass in high-light conditions were shown to be cumulative over the growing period, dependent on the number of transgenes expressed. For example, the increase in overall biomass for lines over-expressing SBPase+ictB was higher than for either SBPase or ictB individually. The same was true for plants over-expressing all three transgenes, with biomass increase being higher in these plants than that observed for either the S, T single- or the SF-double transgene plants. Interestingly, the total dry weight of the transgenic plants correlated well with the relative increases seen in photosynthesis in the young seedlings. These data provide evidence that chlorophyll fluorescence imaging can be used as a robust tool to allow the screening and identification of young seedlings with improved photosynthesis which results in improved yield at maturity.

## Conclusion

In this study, it has been demonstrated that the over-expression of two C3 cycle enzymes leads to an increase in photosynthesis and a cumulative increase in overall biomass yield. It is also shown that over-expression of ictB, a protein proposed to be involved in inorganic carbon transport, in combination with SBPase (ictB and SBPase) or SBPase and FBPA (SBPase, FBPA, and ictB) brought about a further significant improvement in both of these parameters. Although there have been a number of publications with single gene manipulations very little data are available in relation to multiple target manipulation. Importantly, the work here also allowed a direct comparative analysis between the different manipulations, as all of the transgenic and wild-type plants were grown and assessed in parallel, identifying the best manipulations for introduction to crop plants. Although it is still necessary to address the issue of the importance of these manipulations in the field, the approach taken in this study provides strong evidence that multigene manipulation of photosynthesis can form an important part of the strategies to increase crop yield.

## Supplementary data

Supplementary data can be found at *JXB* online.

Supplementary Materials and Methods. A detailed description of construct generation and primers.

Supplementary Fig. S1. Schematic representation of (A) the *A. thaliana* FBPaldolase (B2-FBPaldoA) and (B) the *Synechocystis* PCC 6803 inorganic carbon transporter (B2-ictB) expression vectors; (C) shows the structure of a dual construct for the expression of both FBPaldolase and ictB.

Supplementary Fig. S2. Comparative analysis of wild type and null segragants used in this study.

Supplementary Fig. S3. Complete data set for enzyme assays in plants analysed and Rubisco protein levels in selected lines

Supplementary Fig. S4. Leaf area at 8 d of development.

Supplementary Fig. S5. Complete data set for all transgenic lines grown in high light conditions evaluated at harvest.

Supplementary Fig. S6. Growth analysis of greenhouse-grown wild type (WT) and transgenic lines at 28 d after planting.

Supplementary Fig. S7. Complete data set for all transgenic lines evaluated at 28 d.

Supplementary Fig. S8. Root development of greenhouse-grown wild type (WT) and transgenic lines.

Supplementary Fig. S9. Complete data set for all transgenic lines grown in low light conditions evaluated at harvest.

Supplementary Table S1. The percentage increase over wild type for each parameter measured in low light- (200–350 μmol m^–2^ s^–1^) and high light- (600–1400 μmol m^–2^ s^–1^) grown plants.

Supplementary Data
