# Supplementary material for: Multigene manipulation of photosynthetic carbon assimilation increases CO2 fixation and biomass yield in tobacco
Source: J Exp Bot. 2015 May 8;66(13):4075–90. doi: 10.1093/jxb/erv204 (PMC4473996; doi:10.1093/jxb/erv204)
Supplement: Supplementary Data [file supp_66_13_4075__index.html]

Multigene manipulation of photosynthetic carbon assimilation increases CO2 fixation and biomass yield in tobacco — Multigene manipulation of photosynthetic carbon assimilation increases CO2 fixation and biomass yield in tobacco — Supplementary Data 

# Multigene manipulation of photosynthetic carbon assimilation increases CO2 fixation and biomass yield in tobacco

## Supplementary Data

Data files

**Files in this Data Supplement:**

- Supplementary Data - Supplementary Data
