# Supplementary material for: Multigene manipulation of photosynthetic carbon assimilation increases CO2 fixation and biomass yield in tobacco
Source: J Exp Bot. 2015 May 8;66(13):4075–90. doi: 10.1093/jxb/erv204 (PMC4473996; doi:10.1093/jxb/erv204)
Supplement: Supplementary Data [file supp_erv204_Simkin_et_al_JExBot_Supp_File.pdf]

Multigene manipulation of photosynthetic carbon assimilation increases CO_2 fixation and biomass yield in tobacco

Andrew J. Simkin, Lorna McAusland, Lauren R. Headland, Tracy Lawson, Christine A. Raines*

Supplementary Data

Materials and Methods

Construct Generation

1.1. Fructose-1,6-bisphosphate aldolase (FBPA)

The full-length coding sequence of *fructose-1,6-bisphosphate aldolase* (FBPA: At4g38970) was amplified by RT-PCR using primers AtFB-F (5′caccATggCATCAACCTCACTCCTCAAgg′3) and AtFB-R (5′TCAATAggTgTACCCTTTgACgAACATgC′3). The resulting amplified product was cloned into pENTR/D (Invitrogen, UK) to make pENTR-*AtFBPA* and the sequence was verified and found to be identical. The full-length cDNA was introduced into the pGWB2 gateway vector (Nakagawa *et al*., 2007: AB289765) by recombination from the pENTR/D vector to make pGWB2-AtFBPA (**B2-FB;** (Supplementary **Fig. S1a** available at *JXB* online). cDNA are under transcriptional control of the 35s tobacco mosaic virus promoter, which directs constitutive high-level transcription of the transgene, and followed by the *nos* 3' terminator.

1.2. Inorganic Carbon Transporter B (ictB)

The codon optimised coding sequence of the ictB (YP399376) from *Synechocystis* PCC 6803 linked to the *Brachypodium distachyon Sedoheptulose-1,7-bisphosphatase* transit peptide (XP_003564625) was generated by synthetic synthesis (Epoch Life Science, Inc, USA). The full-length coding sequence (*SBTPictB*) was amplified directly from the synthetic mix using primers ictBf (5′CACCCGGGCAGCAATGGAGACCGTGG′3) and ictBr (5′GATATCCTACATTTTTTCGTCTGAATGCTCGG′3). The resulting amplified product was cloned into pENTR/D (Invitrogen, UK) to make pENTR-*SBPTictB* and the sequence was verified by sequencing. The full-length cDNA was introduced into the pGWB2 gateway vector (Nakagawa *et al*., 2007: AB289765) by recombination from the pENTR/D vectors to make

pGWB2-SBTPictB (**B2-TB;** (Supplementary **Fig. S1b** available at *JXB* online). cDNA are under transcriptional control of the 35s tobacco mosaic virus promoter, which directs constitutive high-level transcription of the transgene, and followed by the *nos* 3' terminator.

1.3. Fructose-1,6-bisphosphate aldolase (FBPA) + Inorganic Carbon Transporter B (ictB)

The full-length coding sequence of *fructose-1,6-bisphosphate aldolase* (FBPA: At4g38970) was introduced into the pDESTOE gateway vector (Booker *et al.,* 2004) by recombination from pENTR-*AtFBPA* to make pDEST-AtFBPA. FBP aldolase was under the control of the promoter FMV (Richins *et al.,* 1987) with the nos terminator. The full-length expression cassette FMV-AtFBPA-NosT was amplified by PCR using primers pDESTF (5'CTGAGA-HindIII-TGAAGGCGGGAAACGACAATCTGATCC'3) and pDESTR (5'CTGAGA-HindIII-AGGCCTTCATAACGTGACTCCCTTAATTCTCC'3). The amplified product was digested with *Hind*III (Promega) and cloned into the unique *Hind*III site of gateway vector pGWB2 (Nakagawa *et al.,* 2007: AB289765) to make vector pASD2 containing the full-length LR site of pGWB2 with the secondary FBPA cassette in reverse orientation (35s-LR cassette-nos-nos-AtFBPA-FMV). The codon optimised coding sequence of the ictB (YP399376) from *Synechocystis* PCC 6803 linked to the *Brachypodium distachyon Sedoheptulose-1,7-bisphosphatase* transit peptide was introduced into the pGWB2 gateway site of vector pASD2 by recombination from the pENTR-*SBPTictB* vectors to make pAS-SBTPictB-FBPA (**FB-TB;** (Supplementary **Fig. S1c** available at *JXB* online). ictB under transcriptional control of the 35s tobacco mosaic virus promoter and FBPA is under control of the FMV promoter. Both promoters drive constitutive high-level transcription of the transgene.

Booker J, Auldridge M, Wills S, Klee HJ, Leyser O. 2004. MAX3 is a carotenoid cleavage dioxygenase required for the synthesis of a novel plant hormone. *Curr. Biol.* **14**, 1–20.

Nakagawa T, Kurose T, Hino T, Tanaka K, Kawamukai M, Niwa Y, Toyooka K, Matsuoka K, Jinbo T, Kimura T. 2007. Development of series of gateway binary vectors, pGWBs, for realizing efficient construction of fusion genes for plant transformation. *Journal of Bioscience and Bioengineering* **104**, 34-41.

Richins RD, Scholthof HB. and Shepard RJ. 1987. Sequence of the figwort mosaic virus DNA (caulimovirus group). *Nucleic Acids Research* **15**, 8451–8466.

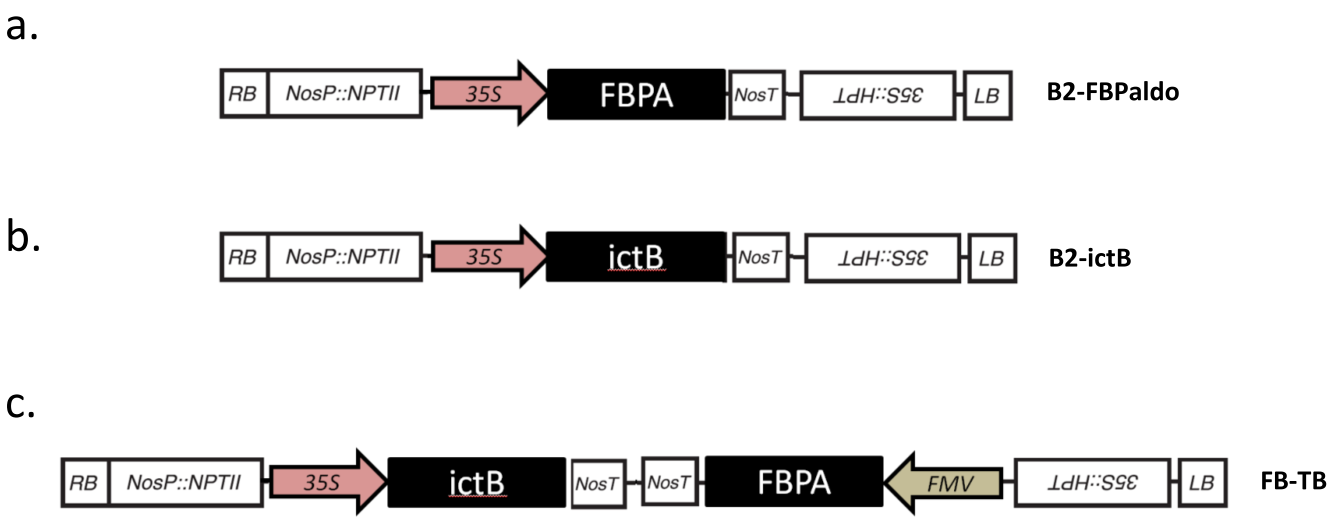

Supp Fig 1. Schematic representation of the A) *A. thaliana* FBPaldolase (B2-FBPaldo) and the B) *Synechocystis* PCC 6803 inorganic carbon transporter (B2-ictB) expression vectors. C) shows the structure of a duel construct for the expression of both FBPaldoalse and ictB. RB, T-DNA right border; Pnos, nopaline synthase promoter; NTP II, neomycin phosphotransferase gene; Tnos, nopaline synthase terminator; P35S, cauliflower mosaic virus 35S promoter, FMV, figwart mosaic virus promoter. Constructs were used to transform wild type tobacco (cv samson) or samson tobacco over-expressing SBPase (Lefebvre et al., 2005)

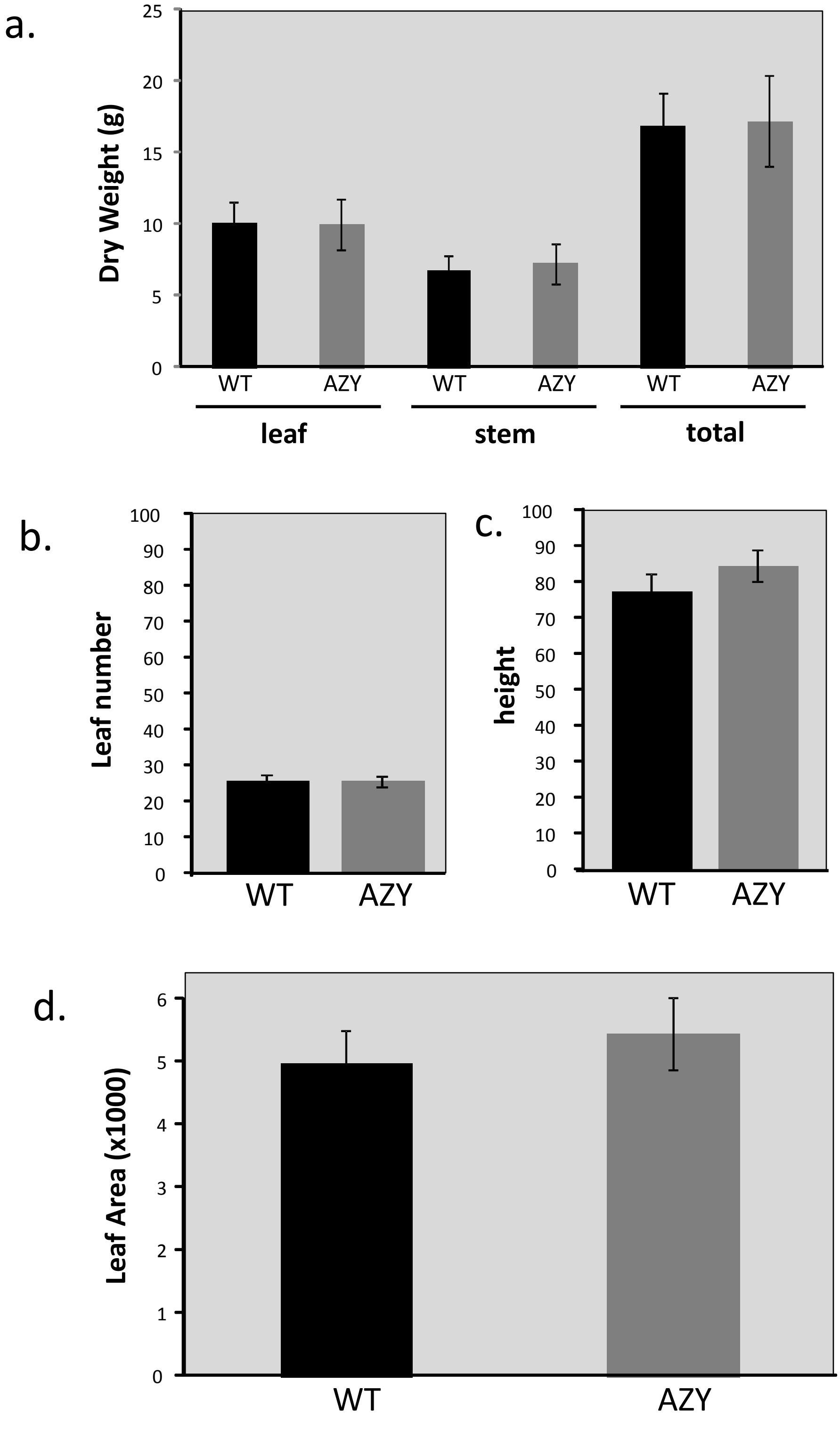

Supp Fig. 2. Comparative analysis of Wild type and null segragants used in this study. WT – wild type plants grown from seed batch. AZY – null segragants recovered from ictB lines segregating population and verified by PCR for non-integration of the transgene. (a) dry weight, (b) leaf number, (c) height of stem, (d) leaf area.

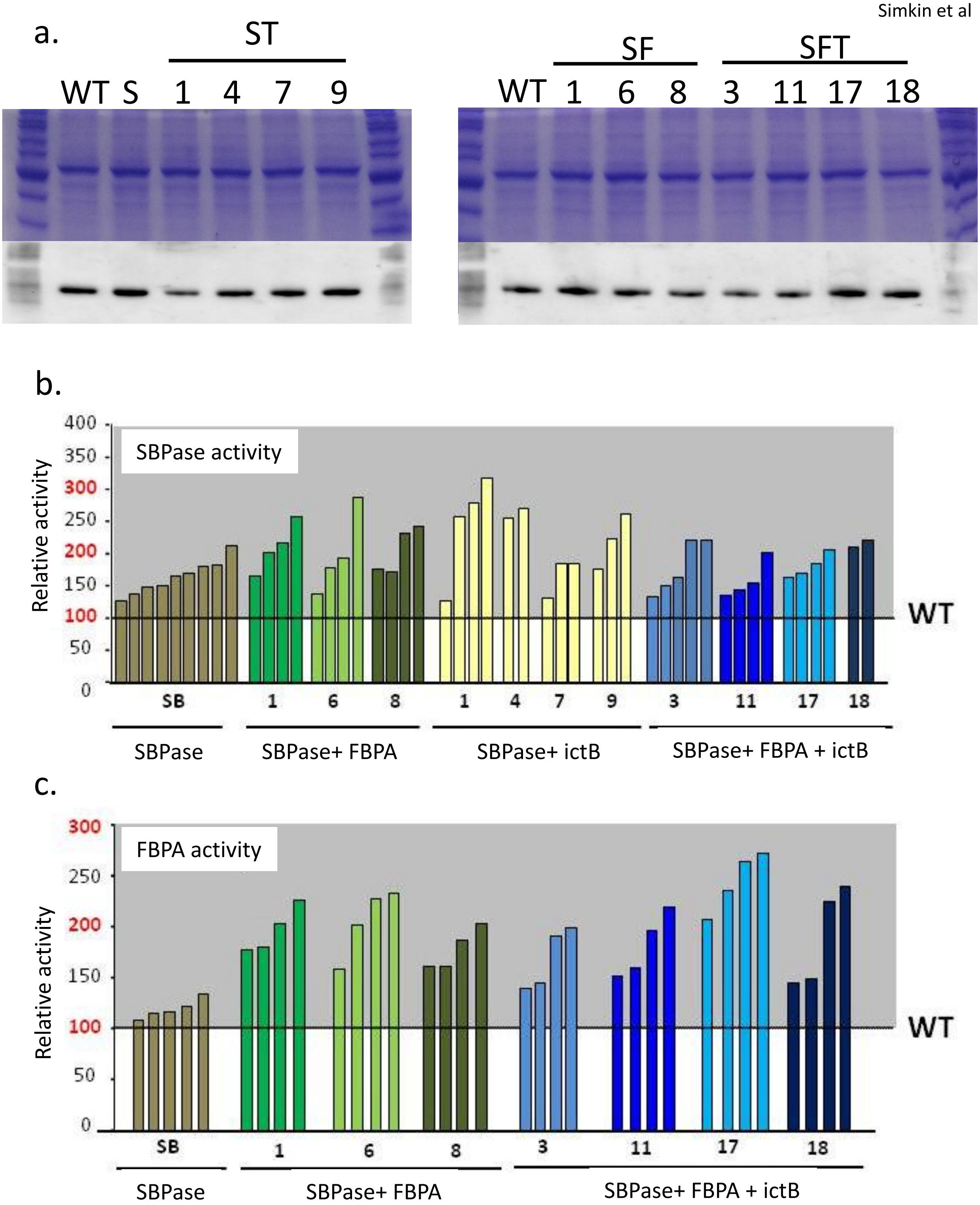

Supp Fig. 3. Complete data set for enzyme assays in plants analysed. (a) rubisco protein levels in selected lines (used for enzyme assays) determined by western blot. Each lane represents protein independently extracted from four individual plants from each line and pooled (b) SBPase and (c) FBPA enzyme activities in fully expanded leaves from individual plants from studied lines.

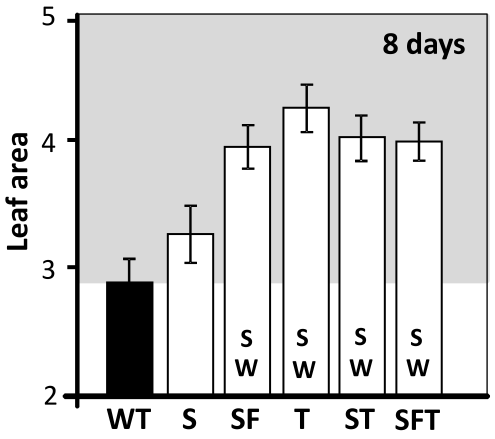

Supp. Fig. 4. Leaf area at 8 days of development. Plant were grown at a maximum 130 µmol m^{-2} s^{-1} light intensity. The data was obtained using 12 to 16 individual plants from 3 to 4 independent transgenic lines. Significant differences (<0.05) between lines is shown using capital letters. Lines over-expressing SBPase (S), SBPase and FBPA (SF), ictB (T), SBPase and ictB (ST) and SBPase, FBPA and ictB (SFT) are represented.

Highlight

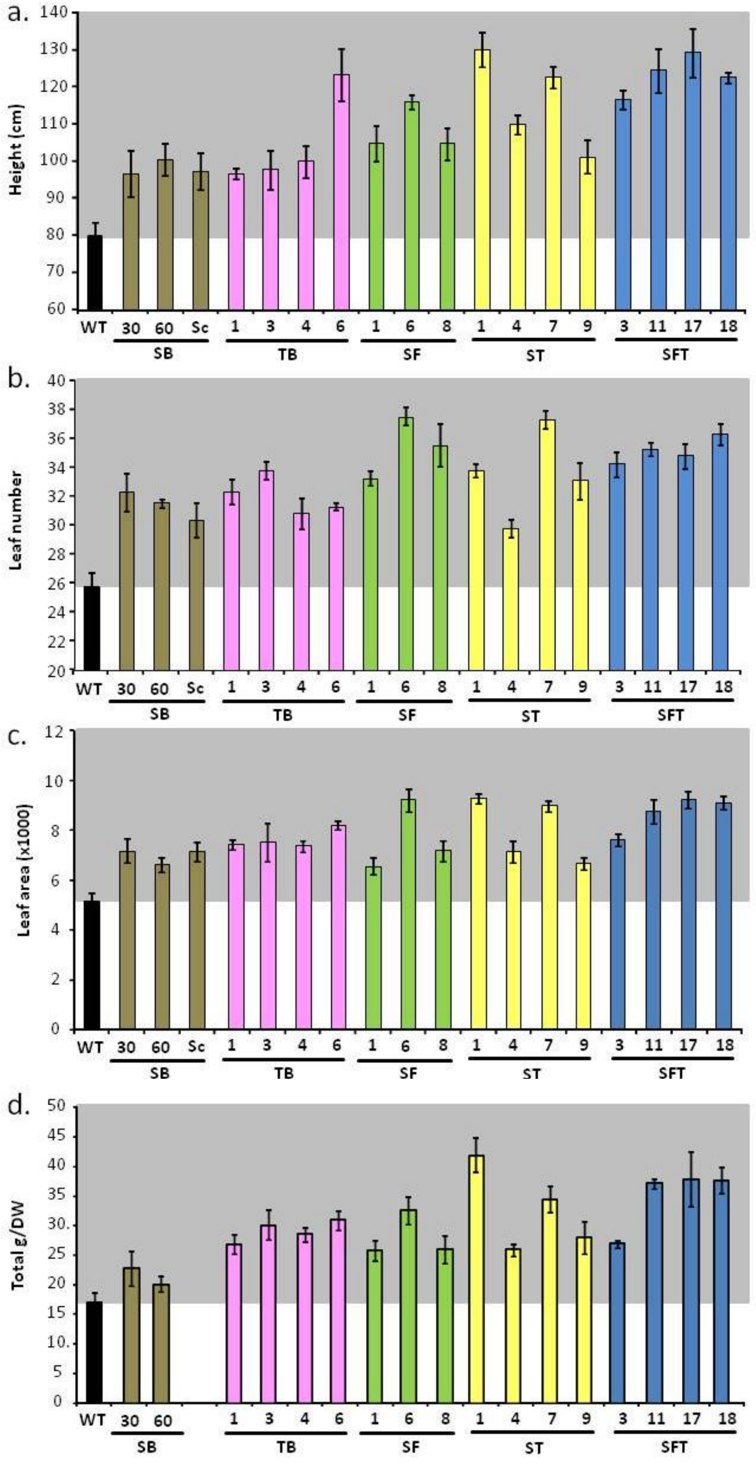

Supp. Fig. 5. Complete data set for all transgenic lines grown in high light conditions evaluated at harvest. (a) height, (b) Leaf number, (c) Leaf Area, (d) Total dry weight. Lines over-expressing SBPase (SB), SBPase and FBPaldolase (SF), ictB (TB), SBPase and ictB (ST) and SBPase, FBPaldolase and ictB (SFT) are represented. Sc represents SBPase lines segregated from lines SF, ST and SFT following transformation. Plants were grown for two weeks at 130 µmol m^{-2} s^{-1} light intensity in long days (12h/12h days) before being transferred to fluctuating and supplemented light (16h/8h days), at maximum 600-1400 µmol m^{-2} s^{-1} light intensity for an additional 4 weeks (6 weeks total). Plants were harvested when the first sign of flower development became apparent. Results are representative of 4 to 5 plants per line

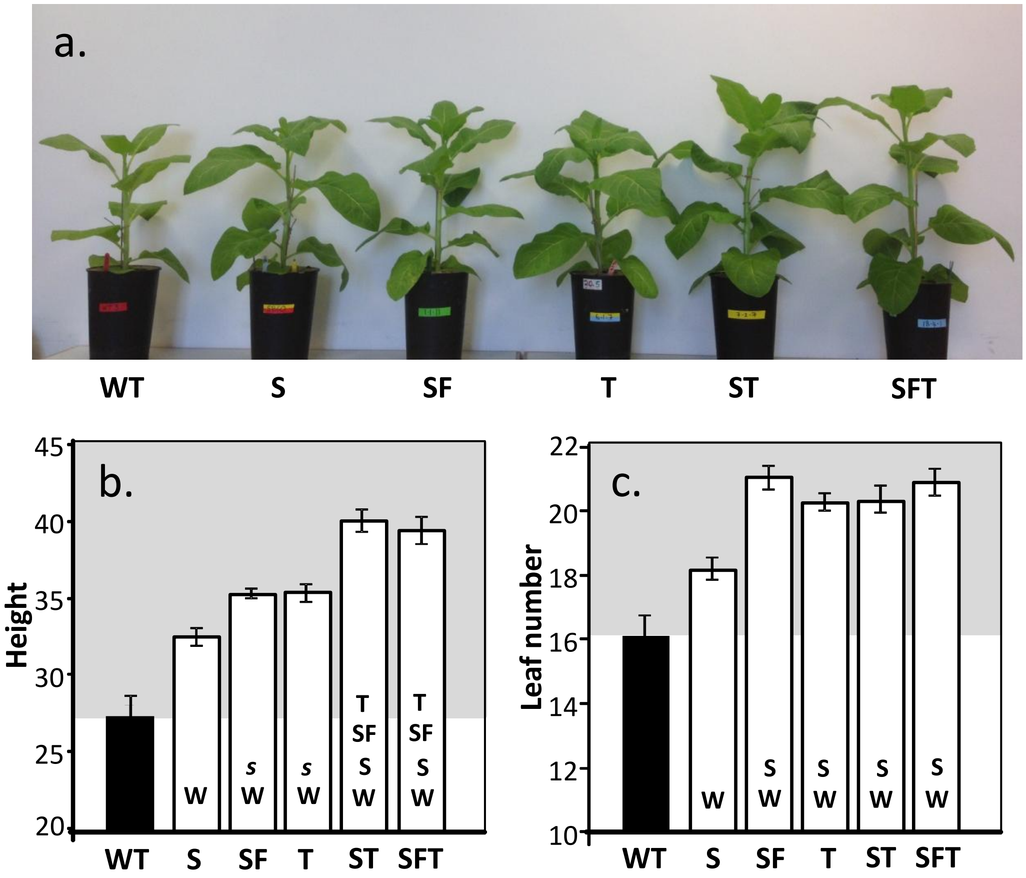

Supp. Fig. 6. Growth analysis of greenhouse grown wild type (WT) and transgenic lines at 28 days after planting. Height and leaf number of plants grown for 2 weeks in a controlled environment (130 µmol m^{-2} s^{-1} light intensity for 12 hours) and transferred to long days, fluctuating and supplemented light, at maximum 600-1400 µmol m^{-2} s^{-1} light intensity for and cultivated for an additional 2 weeks (28 days after potting). (a) plant at time of measurement, (b) height, (c) leaf number. Results are representative of 4 to 6 plants from 3 to 4 individual lines (12-16 plants total). Significant differences (<0.05) are represented as letters indicating if each specific line is significantly different from another. Lower case italic lettering indicates lines are just below significance (>0.05 - <0.1). Lines over-expressing SBPase (S), SBPase and FBPA (SF), ictB (T), SBPase and ictB (ST) and SBPase, FBPA and ictB (SFT) are represented.

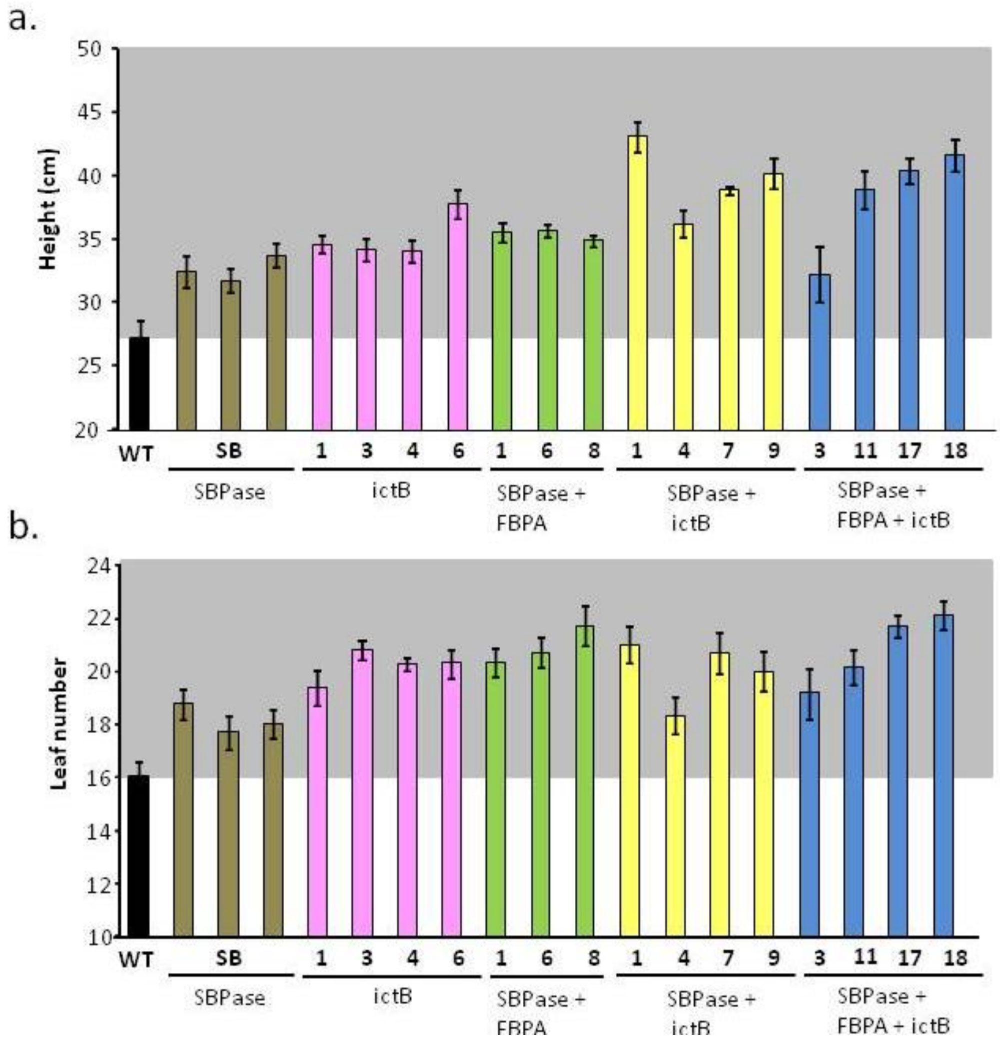

Supp. Fig. 7. Complete data set for all transgenic lines evaluated at 28 days. (a) height, (b) Leaf number, (Lines over-expressing SBPase, SBPase and FBPA, ictB, SBPase and ictB and SBPase, FBPA and ictB (SFT) are represented. Plants were grown for two weeks at 130 µmol m^{-2} s^{-1} light intensity in long days (12h/12h days) before being transferred to fluctuating and supplemented light (16h/8h days), at maximum 600-1400 µmol m^{-2} s^{-1} light intensity for an additional 2 weeks (28 days). Results are representative of 4 to 6 plants per lines.

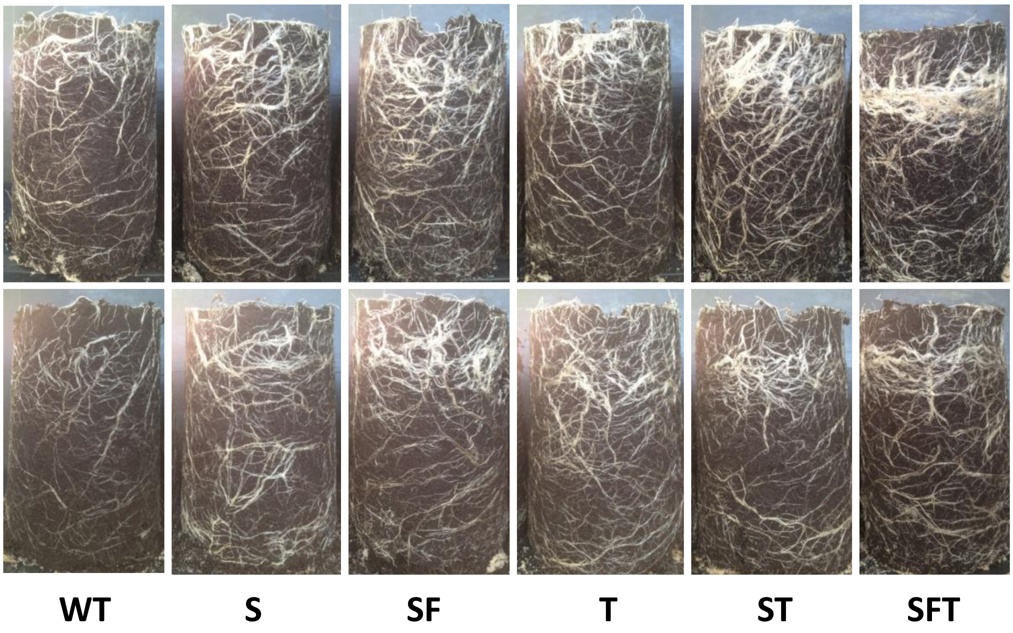

Supp. Fig. 8. Root development of greenhouse grown wild type (WT) and transgenic lines. Lines over-expressing SBPase (S), SBPase and FBPaldolase (SF), ictB (T), SBPase and ictB (ST) and SBPase, FBPaldolase and ictB (SFT) are represented. Plants were grown in long days for 4 weeks, fluctuating and supplemented light, at maximum 650 µmol m^{-2} s^{-1} light intensity. Plants were harvested when the first sign of flower development became apparent. Roots show higher density in transgenic lines compared to wild type consistent with the more advanced development of transgenic lines.

Supplementary Table 1 shows the percentage increase over wild type for each parameter measured in low light (200-350 µmol $m^{-2}s^{-1}$) and high light (600-1400 µmol $m^{-2}s^{-1}$) grown plants.

LOW LIGHT	Characteristics			Biomass		
% increase from WT	Leaf No.	Leaf Area	Height	Leaf	Stem	TOTAL
SBPase	21	35	16	53	51	52
SBPase + FBPaldolase	29	33	17	56	50	54
SBPase + ictB	29	47	31	73	80	76
SBPase + FBPaldolase + ictB	29	46	29	76	82	79

HIGH LIGHT	Characteristics			Biomass		
% increase from WT	Leaf No.	Leaf Area	Height	Leaf	Stem	TOTAL
SBPase	22	36	23	26	25	34
SBPase + FBPaldolase	38	49	36	56	71	62
ictB	24	48	31	58	90	71
SBPase + ictB	30	56	47	80	110	92
SBPase + FBPaldolase + ictB	36	67	54	88	124	103

Lowlight

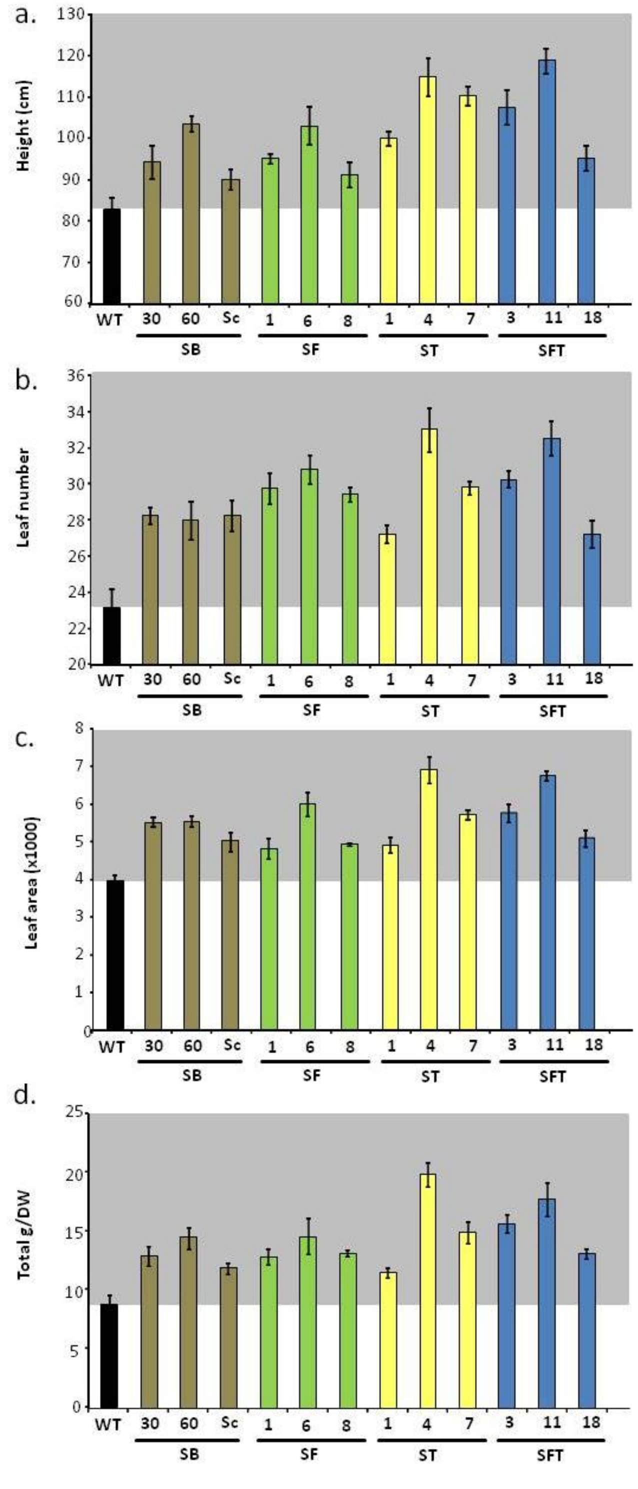

Supp. Fig. 9. Complete data set for all transgenic lines grown in low light conditions evaluated at harvest. (a) height, (b) Leaf number, (c) Leaf Area, (d) Total dry weight. Lines over-expressing SBPase (SB), SBPase and FBPA (SF), SBPase and ictB (ST) and SBPase, FBPA and ictB (SFT) are represented. Sc represents SBPase lines segregated from lines SF, ST and SFT following transformation. Plants were grown for two weeks at 130 µmol m^{-2} s^{-1} light intensity in long days (12h/12h days) before being transferred to fluctuating and supplemented light (16h/8h days), at maximum 200-350 µmol m^{-2} s^{-1} light intensity for an additional 5 weeks (7 weeks total). Plants were harvested when the first sign of flower development became apparent. Results are representative of 4 to 5 plants per line
